# Native mass spectrometry identifies an alternative DNA-binding pathway for BirA from *Staphylococcus aureus*

**DOI:** 10.1038/s41598-019-39398-6

**Published:** 2019-02-26

**Authors:** Jiulia Satiaputra, Louise M. Sternicki, Andrew J. Hayes, Tara L. Pukala, Grant W. Booker, Keith E. Shearwin, Steven W. Polyak

**Affiliations:** 1 School of Biological Sciences, University of Adelaide, Adelaide, South Australia 5005 Australia; 2 School of Physical Sciences, University of Adelaide, Adelaide, South Australia 5005 Australia; 3 Present Address: Harry Perkins Institute of Medical Research, Shenton Park, Western Australia 6008 Australia; 4 Present Address: Faculty of Health and Medical Sciences, Adelaide, South Australia 5005 Australia; 5 Present Address: School of Pharmacy and Medical Sciences, University of South Australia, Adelaide, South Australia 5001 Australia

## Abstract

An adequate supply of biotin is vital for the survival and pathogenesis of *Staphylococcus aureus*. The key protein responsible for maintaining biotin homeostasis in bacteria is the biotin retention protein A (BirA, also known as biotin protein ligase). BirA is a bi-functional protein that serves both as a ligase to catalyse the biotinylation of important metabolic enzymes, as well as a transcriptional repressor that regulates biotin biosynthesis, biotin transport and fatty acid elongation. The mechanism of BirA regulated transcription has been extensively characterized in *Escherichia coli*, but less so in other bacteria. Biotin-induced homodimerization of *E. coli* BirA (*Ec*BirA) is a necessary prerequisite for stable DNA binding and transcriptional repression. Here, we employ a combination of native mass spectrometry, *in vivo* gene expression assays, site-directed mutagenesis and electrophoretic mobility shift assays to elucidate the DNA binding pathway for *S. aureus* BirA (*Sa*BirA). We identify a mechanism that differs from that of *Ec*BirA, wherein *Sa*BirA is competent to bind DNA as a monomer both in the presence and absence of biotin and/or MgATP, allowing homodimerization on the DNA. Bioinformatic analysis demonstrated the *Sa*BirA sequence used here is highly conserved amongst other *S. aureus* strains, implying this DNA-binding mechanism is widely employed.

## Introduction

The biotin retention protein A (BirA, also known as biotin protein ligase) is responsible for maintaining biotin homeostasis in many bacteria. BirA is a bi-functional protein capable of both enzymatic protein biotinylation as well as serving as a biotin-controlled transcriptional repressor that regulates the expression of the biotin biosynthesis operon (reviewed^1,2^). By combining both activities in a single protein, BirA is uniquely placed as the key regulator of biotin metabolism. *E. coli* BirA (*Ec*BirA) has been thoroughly investigated through genetic, biochemical and structural biology studies^3–11^. These have provided powerful insights into the maintenance of biotin homeostasis. *Ec*BirA binds to its ligands, biotin and ATP, in an ordered manner^6,12^. Conformational changes induced by biotin binding create the pocket necessary for ATP to bind. ATP binding initiates the synthesis of biotinyl-5′-AMP, which serves as both a reaction intermediate for biotin ligation as well as a co-repressor. The BirA:biotinyl-5′-AMP complex, known as the holo-enzyme, is then available for two alternative activities: either as a biotin protein ligase when there is a substrate available for protein biotinylation or when demand for biotin is low, as a transcriptional repressor through homodimerization^13^. The transcriptional repressor function of *Ec*BirA involves a co-operative interaction between two *Ec*BirA subunits and an inverted palindromic repeat sequence present in the promoter of the biotin biosynthetic operon (*bioO*). Homodimerization of the holo-enzyme complex (*K*_D_^2-1^ = 1–6 × 10^−6^ M^14,15^) is a pre-requisite for DNA binding^4,5,16,17^. The unliganded enzyme (i.e. apo-*Ec*BirA) does not dimerize at physiological concentrations (*K*_D_^2-1^ = 1 × 10^−3^ M^14^) and is unable to bind DNA^9,17^. Mutagenesis studies have further highlighted the importance of homodimerisation in the DNA binding mechanism. Amino acid substitutions that promote homo-dimerization display increased affinity for DNA and function as super-repressors^18,19^. Conversely, replacing key amino acids in the interface between the two *Ec*BirA subunits disrupts dimerization, and eliminates DNA-binding activity^7,15,17,20,21^. One well-characterized example is *Ec*BirA-R119W^20,22^ (Supporting Fig. S1A) that is essentially monomeric in solution (*K*_D_^2-1^ = 2 × 10^−2^ M^17^). This dimerization-impaired mutant thus mimics the association state of apo-*Ec*BirA.

*S. aureus* BirA (*Sa*BirA) shares many features with its *E. coli* homologue. The two proteins have similar tertiary structures despite limited sequence conservation (24% identity, 42% similarity, Supporting Fig. S1B). Both proteins undergo analogous conformational changes that define the ordered ligand binding mechanism^23–25^. Like *Ec*BirA, *Sa*BirA is a biotin-dependent transcriptional repressor of biotin biosynthesis^26,27^. Unlike *Ec*BirA however, *Sa*BirA regulates expression of additional operons - the substrate specific subunit of the biotin transporter (encoded by *bioY*), and the *yhfS*-*yhfT* operon encoding putative homologs of acetyl-CoA acetyl transferase and long-chain fatty acid-CoA ligase, respectively^26,28^. Holo-*Sa*BirA binding directly to the promoter sequence of the biotin biosynthesis operon (*SabioO*) has recently been demonstrated by us^24,26,29^, and others^27^. Estimates of the dissociation constant (*K*_D_), based on polyacrylamide gel-based electrophoretic mobility shift assays (EMSA), suggest a 6-fold difference in the affinity for DNA between apo and holo-*Sa*BirA (apo 649 nM, holo 108 nM^29^). In support of these results, fluorescence anisotropy experiments performed in solution suggest the holo-protein binds 63-fold tighter to DNA than the apo-protein (apo 5 μM, holo 83 nM^27^). The modest differences in affinity for the apo enzyme are likely due to polymorphisms in the *Sa*BirA sequences used in the two studies. Despite the intrinsic DNA-binding activity of the apo enzyme, *Sa*BirA has been shown to function as a biotin-dependent transcriptional repressor^26,27^. These observations are significantly different to *Ec*BirA, where apo-protein is devoid of DNA-binding activity.

In this study, the homodimerisation and DNA-binding activities of *Ec*BirA and *Sa*BirA were investigated using *in vivo* reporter assays and *in vitro* biochemical analysis. For the first time, native mass spectrometry was utilized to study the self-association and DNA binding functions of BirA. Nano-electrospray ionization-mass spectrometry (nESI-MS) performed under native conditions is an ideal technique for studying BirA as the soft ionization employed maintains non-covalent interactions during the transit from solution to gas phase^30^. This permitted the direct detection of biotinyl-5′-AMP binding to BirA, as well as quantitating the stoichiometry of the protein:DNA complexes. Engineered protein mutants designed to disrupt homodimerization, namely *Ec*BirA-R119W and the *S. aureus* equivalent *Sa*BirA-F123G (Supporting Fig. S1), were also employed to address the requirement for protein dimerization on DNA-binding (*in vitro*) and transcriptional repression (*in vivo*). Together these data revealed that monomeric *Sa*BirA is competent to bind DNA and, once bound, can recruit a second protein subunit. This new pathway is not evident with *Ec*BirA. Bioinformatic analysis identified that the *Sa*BirA enzyme used in this study, from the methicillin and vancomycin resistant *S. aureus* strain Mu50, is the prototypical BirA amongst the *S. aureus* species. We propose that this DNA binding mechanism may be widely used amongst *S. aureus*.

## Results

### Biotin-induced repression of transcription

**Figure 1:**
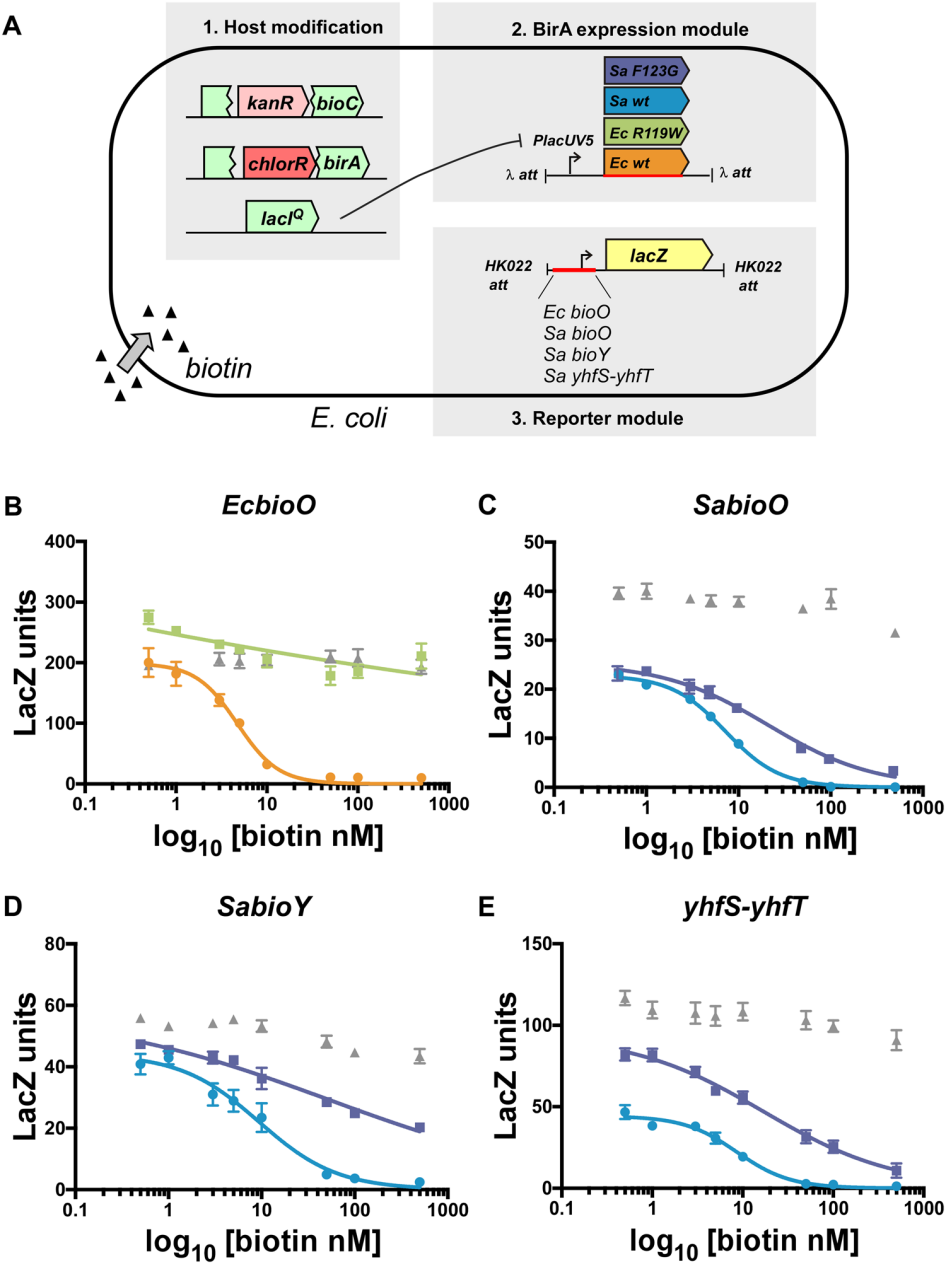
*In vivo* β-galactosidase assays to measure biotin-induced repression. (**A**) Overview of *E. coli* reporter strains containing chromosomally integrated reporters. (**B**–**E**) β-galactosidase assays revealing biotin-induced repression by *Ec*BirA (orange circles), *Ec*BirA-R119W (green squares), *Sa*BirA (blue circles) or *Sa*BirA-F123G (purple squares). Target promoter sequences investigated were (**B**) *EcbioO*, (**C**) *SabioO*, (**D**) *SabioY* or (**E**) *SayhfS-yhfT*. Strains with no integrated BirA served as controls (grey triangles). A further control lacking an integrated promoter was used to measure the background *lacZ* activity at each biotin concentration (≤10 units), which has been subtracted to give values shown in the graphs. Error bars denote S.E.M from independent biological replicates (n = 6).

Apo-*Sa*BirA has been shown to bind DNA *in vitro*^27,29^. We sought to address if the non-liganded protein possessed repressor activity in an *in vivo* assay of gene expression. As it is technically challenging to achieve the apo-state *in vivo*, we employed the *Sa*BirA-F123G mutant that impairs homo-dimerization and, therefore, mimics the monomeric properties of the non-liganded protein^29^. Wild-type *Ec*BirA and *Sa*BirA, and the dimerization disrupted *Ec*BirA*-*R119W mutant^31^, were used as controls. Biotin-induced repressor activity was measured using a bacterial reporter system we have previously described^26^ and summarized in Fig. 1A. Briefly, a *birA* gene under the control of the pLac-UV5 promoter was site-specifically integrated into the lambda phage attachment site (attB) in the chromosome of a biotin auxotrophic strain of *E. coli* harboring a mutant BirA that is unable to bind DNA^26^. The host strain also produces lac repressor from the strong LacI^q^ promoter, allowing us to repress expression of the BirA variants down to the low concentrations necessary for a physiologically relevant assay. A second construct containing a BirA-target promoter fused to a *lacZ* reporter gene was then integrated into the HK022 attB phage attachment site. Here, the promoters for *bioO* from both *E. coli* (*EcbioO*) and *S. aureus* (*SabioO*) were analyzed alongside promoters for the *bioY* and the *yhfS-yhfT* operon in *S. aureus*^26^. Biotin-mediated gene control was then assayed using β-galactosidase activity as a readout for gene expression. The effect of exogenous biotin on these genetic circuits, engineered into the genome of a strain unable to synthesis its own biotin, was measured by the addition of biotin to the growth media.

The *in vivo* reporter assay was developed to reconstitute the biotin-induced transcriptional repressor activity of BirA. Previous studies have suggested that *E. coli* contains ≈10–20 molecules of BirA per cell^32^. Therefore, the assay was designed to minimize artefactual binding to DNA due to the overexpression of BirA. Our previous work has demonstrated that the level of lac repressor provided from a single copy pLacI^q^-lacI module is able to very significantly, though not completely, repress a single copy plac-UV5 promoter module^33^. As expected, biotin-regulated repression of β-galactosidase was observed when IPTG was omitted from the growth media - implying the low level of leaky expression from the repressed lacUV5 promoter provided sufficient *Sa*BirA for activity. Conversely, addition of 10 μM IPTG induced much higher expression of *Sa*BirA, as detected by Western blot, and also abolished biotin-regulated control of the reporter (Supporting Fig. S2). Similarly, the level of *Ec*BirA expression obtained in the absence of IPTG was shown to progressively inhibit β-galactosidase expression with increasing levels of biotin present in the growth media, as expected (Fig. 1B). Hence, all *in vivo* experiments were performed in the absence of IPTG (Fig. 1B–E).

The concentrations of biotin required to achieve half-maximum repression at equilibrium (*R*_bio_) were calculated for each protein (Table 1). For *E. coli*, half maximal repression was achieved with 3.8 nM biotin. Likewise, biotin-induced repression was similar for all three *S. aureus* target promoters, with 7–10 nM biotin required for the same level of response (Table 1). Consistent with previous literature, the *Ec*BirA-R119W mutant that lacks homo-dimerisation activity^22,31^ was also devoid of repressor activity *in vivo* (Fig. 1B, green symbols) yielding similar levels of β-galactosidase expression as the no-repressor control (Fig. 1B, grey symbols). In contrast, *Sa*BirA-F123G repressed activity against all three target promoters in a biotin-dependent manner (Fig. 1C–E, purple curves). For *SabioO* and *SayhfS-yhfT*, the *R*_bio_ values were 2.6-fold and 2.3-fold higher for *Sa*BirA-F123G than wild-type protein, respectively (Table 1) (p = 0.07 WT *vs* F123G for *SabioO*; p = 0.2, WT *vs* F123G for *SayhfS-yhfT*). This modest decrease in repressor activity is consistent with a 3-fold higher *K*_M_ for biotin that has been reported for *Sa*BirA-F123G relative to the wild-type enzyme^29^. For *SabioY*, an accurate estimate of *R*_bio_ was not achieved as 500 nM of biotin was insufficient to completely inhibit expression down to the background level necessary to generate a concentration-dependent repression curve. At the lowest biotin concentration tested (1 nM), β-galactosidase expression from the *SabioO* and *SayhfS-yhfT* promoters was significantly lower than the corresponding no-repressor controls (Fig. 1C,E, wild-type blue and *Sa*BirA-F123G purple *vs* control grey symbols) implying both the wild-type and *Sa*BirA-F123 mutant proteins partially occupied the DNA under these conditions. Together this data suggests monomeric *Sa*BirA (a mimic of the apo-state) is capable of repressing its target genes, which is not evident with *Ec*BirA.

**Table 1:** *In vivo* equilibrium binding constants for biotin-induced transcriptional repression.

Promoter	Repression constant (*R*_bio_) nM	
	Wild-type	Dimerization-impaired mutant
*EcbioO*	3.8 ± 0.6	>500
*SabioO*	7.0 ± 0.3	18.6 ± 5.6
*SabioY*	6.1 ± 2.6	>500
*SayhfS-yhfT*	10.0 ± 2.0	22.6 ± 9.7

The concentration of biotin required to achieve half-maximum repression (*R*_bio_) at equilibrium in the β-galactosidase reporter assay was calculated from the data presented in Fig. 1B–E. Data was fitted using the one-site specific binding equation Y = Bmax*X^h^/(R_bio_^h^ + X^h^), where Y = *LacZ* units, X = biotin concentration (nM), Bmax = maximum binding (*LacZ* units) and h = Hill-slope (BirA binding as a homodimer, h = 2). Datum is from six independent experiments.

### *In vitro* analysis of apo and holo-BirA oligomeric states

**Figure 2:**
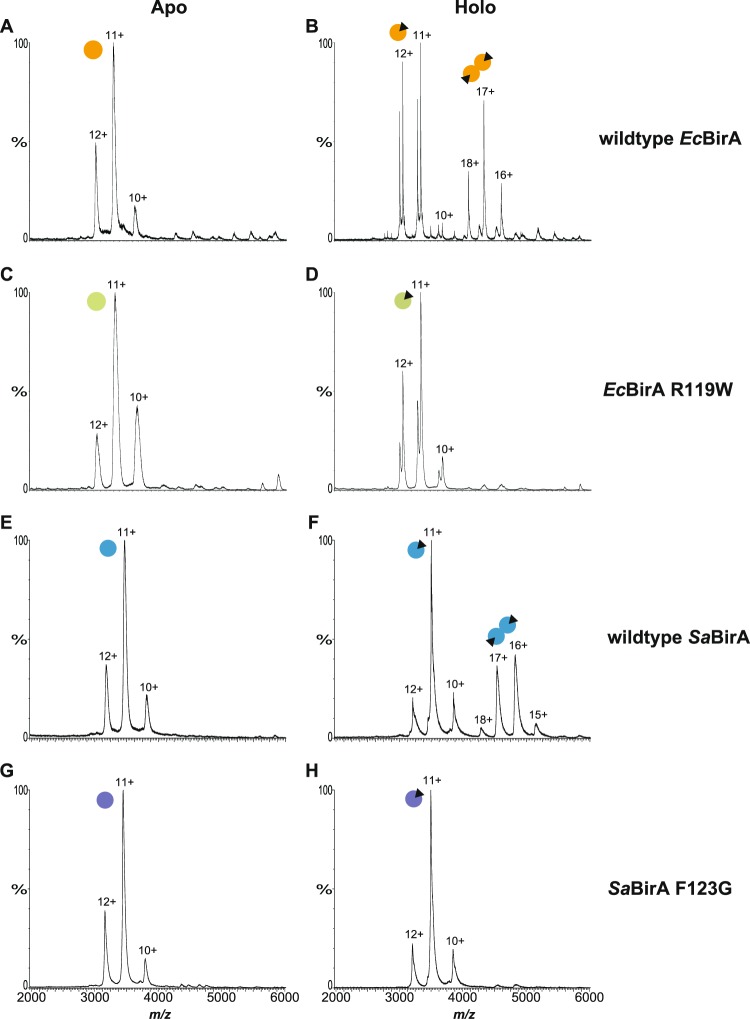
Native nano-electrospray ionization-mass spectrometry determination of the oligomeric state of wild-type BirA and dimerization-impaired mutants. nESI-MS spectra of (**A**) apo-*Ec*BirA, (**B**) holo-*Ec*BirA, (**C**) apo-*Ec*BirA-R119W, (**D**) holo-*Ec*BirA-R119W, (**E**) apo-*Sa*BirA, (**F**) holo-*Sa*BirA, (**G**) apo-*Sa*BirA-F123G and (**H**) holo-*Sa*BirA-F123G. Peaks revealing the oligomeric state of the proteins are marked by the sphere symbols and are annotated with the corresponding charge states. Monomers are denoted by a single sphere, homodimers are shown by two joined-spheres and the presence of biotinyl-5′-AMP is represented with a black triangle.

The molecular basis of the unexpected repressor activity observed with *Sa*BirA-F123G was further investigated using biochemical assays. Wild-type *Ec*BirA and *Sa*BirA were purified in their apo-forms alongside the two dimerization-impaired mutant proteins *Ec*BirA-R119W and *Sa*BirA-F123G. Removal of biotin from the recombinant proteins was achieved by incubating cell lysates (containing over-expressed protein) with streptavidin-Sepharose to remove excess biotin, before incubation with a biotin-accepting substrate protein to facilitate protein biotinylation and the concomitant loss of biotinyl-5′-AMP from the BirA active site prior to IMAC purification. The apo-state of the four proteins was addressed using two alternative techniques that independently confirmed that all preparations were devoid of any co-purified biotinyl-5′-AMP; a streptavidin-blot method we have previously described^29^ (Supporting Fig. S3) and native nESI-MS that has not been employed previously for the study of BirA. The Streptavidin-blot assay failed to detect biotinyl-transferase activity for all four proteins, as expected for an apo-enzyme. In support, the masses measured by nESI-MS for the apo-preparations were as expected for monomeric BirA devoid of ligand (Fig. 2A,C,E and G and Supporting Table S4). We confirmed that the mild conditions employed with the native mass spectrometry studies allowed the direct measurement of BirA in complex with biotinyl-5′-AMP, as well as the oligomeric state of holo-BirA (Fig. 2B,D,F and H). The addition of biotin and MgATP to *Ec*BirA and *Sa*BirA resulted in the detection of two species (Fig. 2B,F), consistent with those expected for the monomeric proteins bound to the reaction intermediate biotinyl-5′-AMP (*Ec*BirA, measured 36771 Da, theoretical molecular mass 36765 Da: *Sa*BirA, measured 38470 Da, theoretical molecular mass 38466 Da) and the dimeric form of these ligand-bound complexes (*Ec*BirA, measured 73559 Da, theoretical molecular mass 73530 Da: *Sa*BirA: measured 76925 Da, theoretical molecular mass 76931 Da). The nESI-MS data confirmed that *Ec*BirA and *Sa*BirA possessed biotinyl-5′-AMP synthetase activity, and the enzyme:biotinyl-5′-AMP complex was stable following their exchange into the volatile 200 mM ammonium acetate pH 6.85 buffer required for mass spectrometry. Incubation of *Ec*BirA-R119W and *Sa*BirA-F123G with biotin and MgATP yielded single species with masses consistent with monomers in complex with biotinyl-5′-AMP (*Ec*BirA-R119W, measured 36783 Da, theoretical molecular mass 36795 Da: *Sa*BirA-F123G, measured 38381 Da, theoretical molecular mass of 38376 Da). There was no evidence of biotin-induced homodimers for either mutant protein (Fig. 2D,H). This demonstrated that whilst *Ec*BirA-R119W and *Sa*BirA-F123G retained biotinyl-5′-AMP synthetase activity, both had abolished in-solution dimerization activity.

### Apo and dimerization-impaired *Sa*BirA are competent to bind DNA

**Figure 3:**
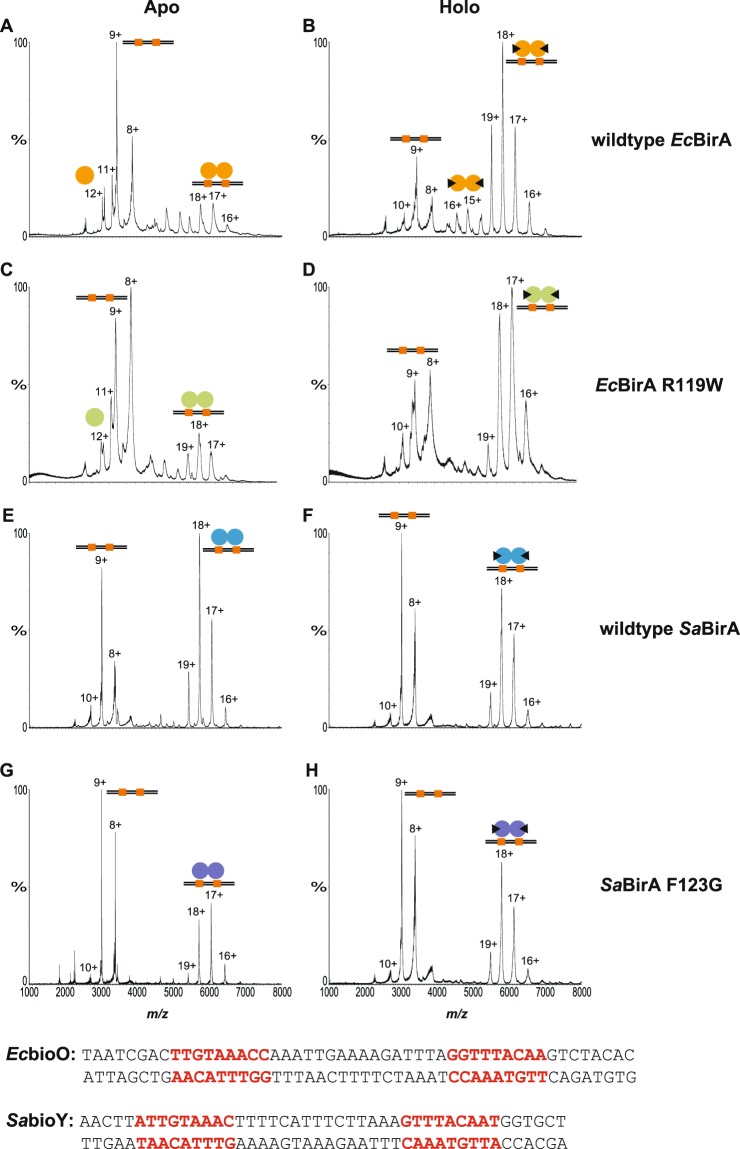
Native nano-electrospray ionization-mass spectrometry analysis of BirA binding to DNA. nESI-MS spectra demonstrating the protein:DNA interaction involving (**A**) apo-*Ec*BirA, (**B**) holo-*Ec*BirA, (**C**) apo-*Ec*BirA-R119W and (**D**) holo-*Ec*BirA-R119W bound to *EcbioO* and (**E**) apo-*Sa*BirA, (**F**) holo-*Sa*BirA, (**G**) apo-*Sa*BirA-F123G and (**H**) holo-*Sa*BirA-F123G bound to *SabioY*. Monomers are denoted by a single sphere, homodimers are shown by two joined-spheres and the presence of biotinyl-5′-AMP is represented with a black triangle. Black lines represent double stranded oligonucleotides containing two BirA binding sites (orange). Peaks corresponding to the major species are annotated with their charge state. The DNA sequences of the oligonucleotides used in the assay are shown below with the two half sites highlighted in red.

Native nESI-MS (Fig. 3) was employed to probe the DNA binding activities of *Ec*BirA and *Sa*BirA. Oligonucleotides containing the recognition sequences from the *EcbioO* and *S. aureus bioY* promoters were employed for the nESI-MS analysis. DNA:protein complexes were formed in 200 mM ammonium acetate pH 6.9 buffer by incubating BirA with the double-stranded oligonucleotides on ice for at least one hour prior to mass spectrometry analysis. A comparison of the theoretical and measured molecular masses for the complexes analysed in this study is shown in Supporting Table S5. The specificity of the BirA:DNA interaction was initially confirmed using an oligonucleotide lacking BirA operators. Neither native nESI-MS nor EMSA detected *Sa*BirA binding to an oligonucleotide with the two BirA half sites mutated to random sequences (Supporting Fig. S4).

**Figure 4:**
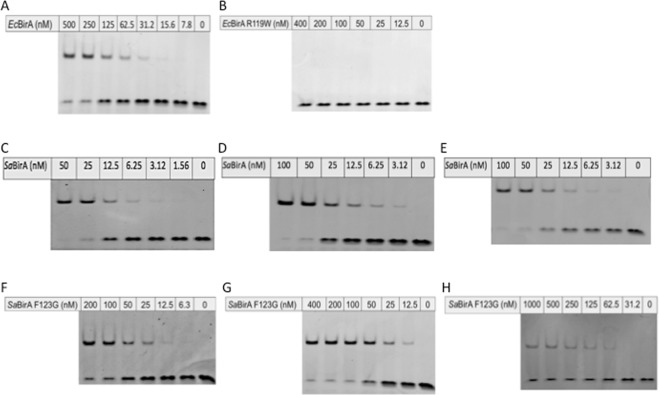
Electrophoretic mobility shift assays. EMSA of the interaction between *EcbioO* and (**A**) *Ec*BirA or (**B**) *Ec*BirA-R119W. Also shown is *Sa*BirA binding to (**C**) *SabioO*, (**D**) *SabioY* or (**E**) *SayhfS-yhfT* and *Sa*BirA-F123G binding to (**F**) *SabioO*, (**G**) *SabioY* and (**E**) *SayhfS-yhfT*. Concentrations of enzyme used in the binding reactions are indicated. Panels C, D and E showing wild type *Sa*BirA binding are from^26^. Full length gels are presented in Supporting Fig. S5.

For holo-*Ec*BirA, the predominant species had a mass consistent with two subunits of *Ec*BirA bound to DNA. For the apo-*Ec*BirA protein, this species was seen only with weak intensity (Fig. 3A,B). For *Ec*BirA-R119W (at a protein concentration of 10 μM) biotinyl-5′-AMP also induced a complex comprising two protein subunits on DNA, a species which was only observed with a weak intensity for apo-*Ec*BirA-R119W (Fig. 3C,D). At equilibrium, the majority of the apo-wild-type *Ec*BirA and *Ec*BirA-R119W was present as monomeric protein, not in complex with DNA. Consistent with the nESI-MS datum, DNA binding was also observed in an EMSA for wild-type holo *Ec*BirA (Fig. 4A). However, holo-*Ec*BirA-R119W mutant binding to DNA could not be measured by EMSA, even at 400 nM BirA (Fig. 4B).

For the *Sa*BirA, evidence of two protein subunits in complex with DNA was observed for wild-type and mutant *Sa*BirA-F123G, in both apo and holo-states (Fig. 3E–H). In both apo-samples minimal free protein was observed, implying most of the *Sa*BirA was in a complex with DNA. This was in contrast to apo-*Ec*BirA where free protein was readily detected. This datum was consistent with EMSA results where both holo-wild-type and holo-*Sa*BirA-F123G were competent to bind DNA (Fig. 4C–H). The EMSA analysis also revealed the holo-wild-type *Sa*BirA binds all three target promoters at similar affinities (Fig. 4C–E). However, the interaction between holo-*Sa*BirA-F123G and *Sa* *yhfS*-*yhFT* was clearly weaker compared to the *SabioO* and *SabioY* operator sequences (Fig. 4E–G) which is likely due to a base pair mismatch in the BirA binding site^26^. Together these findings highlight a key difference between the *E. coli* and *S. aureus* BirAs – monomeric (i.e. apo) *Sa*BirA is capable of stable binding to DNA.

### Ordered assembly of BirA on DNA

**Figure 5:**
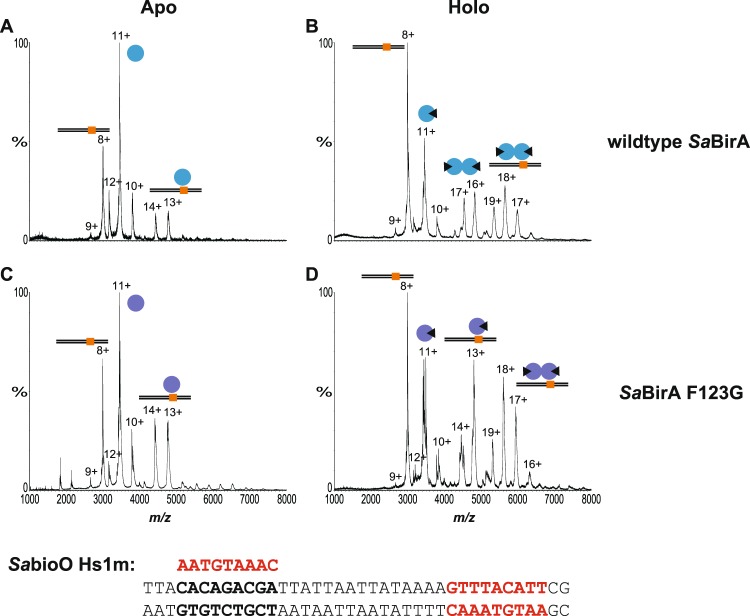
Native nano-electrospray ionization-mass spectrometry analysis of *Sa*BirA binding to DNA containing one binding site. nESI-MS spectra demonstrating the interaction between *SabioO* oligonucleotide containing only one BirA binding half site with (**A**) apo-*Sa*BirA, (**B**) holo-*Sa*BirA, (**C**) apo-*Sa*BirA-F123G and (**D**) holo-*Sa*BirA-F123G. Monomers are denoted by a single sphere, homodimers are shown by two joined-spheres and the presence of biotinyl-5′-AMP is represented with a black triangle. Black lines represent double stranded oligonucleotides containing one BirA binding site (orange). The charged states of the major species are annotated on the spectra. The DNA sequences of the oligonucleotides used in the assay are shown below with the wild-type half sites highlighted in red and the mutated half site in bold text.

The ordered assembly of *Sa*BirA on DNA was finally addressed by native nESI-MS using a *SabioO* oligonucleotide that contained a single half site (Fig. 5, Supporting Table S7). The apo-*Sa*BirA sample revealed a species consistent with one monomer bound to DNA with a charge state distribution centered around 13+ and 14+ (Fig. 5A). However, no DNA-bound dimer was observed. In contrast, holo-*Sa*BirA revealed free dimer and a species consistent with two holo-*Sa*BirA subunits in complex with DNA (Fig. 5B). Therefore, under high biotin conditions holo-*Sa*BirA can dimerise prior to binding DNA, such as occurs with *Ec*BirA. Like the wild-type protein, apo-*Sa*BirA-F123G was also competent to bind DNA as a single BirA subunit (Fig. 5C). Holo-*Sa*BirA-F123G was able to bind as a single subunit bound to DNA and well as a complex of two protein subunits bound to the mutated *SabioO* oligonucleotide (Fig. 5D). This datum suggests that once a single subunit of *Sa*BirA is bound, a second subunit can subsequently bind and stabilize the complex. This DNA-induced protein dimerization can proceed despite the inability of *Sa*BirA-F123G to dimerise in solution. EMSA analysis did not detect holo-*Sa*BirA binding to the mutated oligonucleotide containing only a single BirA binding site (Supporting Fig. S6). It is likely that the experimental conditions of an EMSA, whereby a protein:DNA complex must endure slow (minutes to hours) electrophoretic separation, may not favour the relatively short lived interactions that native nESI-MS can measure on the millisecond timescale. Thus, the native nESI-MS data strongly suggests a pathway where *Sa*BirA dimers can assemble on the DNA one subunit at a time.

### Bioinformatic analysis of *S. aureus* BirA sequences

**Figure 6:**
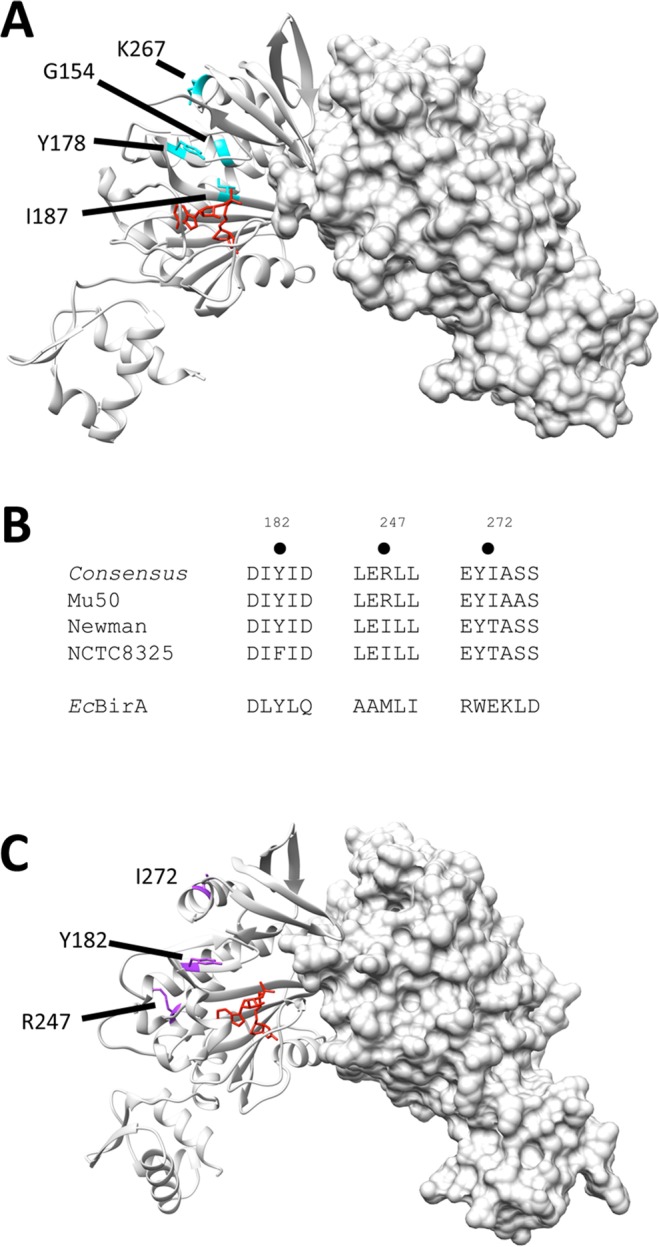
*Sa*BirA sequence analysis. (**A**) Crystal structure of *Ec*BirA [PDBID 2EWN^6^] highlighting amino acids that influence homodimerization when mutated. (**B**) 26 non-redundant *Sa*BirA sequences were aligned to determine the consensus sequence (see Supporting Fig. S7 for full alignment). Differences between *S. aureus* strains Mu50, Newman and NCTC 8325 are shown here. The *Ec*BirA sequence is also aligned (below) for comparison. C. Crystal structure of *Sa*BirA [PDBID 4DQ2^23^] showing the position of amino acids that are not conserved between *S. aureus* strains Mu50, Newman and NCTC8325.

Several groups have now reported studies characterising BirA from *S. aureus*^24,25,27,29,34^. However, these studies have used genes encoding *birA* that have been cloned from different strains bearing different sequences making it difficult to directly compare the findings between studies. It was necessary to identify a consensus *Sa*BirA sequence as detailed studies on the *E. coli* homologue have demonstrated that substitution of certain amino acids can disrupt networks of long-range bonding interactions that can effect homodimerization, DNA binding and transcriptional repression despite not being localized to the dimer interface nor DNA binding domain (Fig. 6A)^18,19,21,35^. To identify the most conserved BirA homolog amongst *S. aureus*, BirA protein sequences were obtained using available genomic datum. A multiple sequence alignment was performed upon 26 non-redundant *Sa*BirA sequences (Supporting Fig. S7), including Mu50 (used in this study and in^24,29,34^), Newman (used in the experiments reported in^25,27^) and the well-studied NCTC 8325 strain. The alignment indicated that the sequence of Mu50 BirA is highly represented amongst the *S. aureus* genomes analysed. The Mu50 sequence differs from both the Newman and NCTC 8325 strains, specifically at positions 247 (R247 in Mu50, I247 in Newman and NCTC 8325) and 272 (I272 in Mu50, T272 in Newman and NCTC 8325) (Fig. 6B,C). Out of the 26 unique sequences analysed, 20 possessed arginine at 247 (conserved with Mu50) and 22 species had isoleucine at position 272 (conserved with Mu50). These residues are located within the central catalytic domain of *Sa*BirA, within α-helical structures (R247 in α-helix 8 and I272 in α-helix 9, Supporting Fig. S8). Neither residue is implicated in interactions with the substrates, biotin or ATP, nor are they located close to the dimerization interface. However, it is possible these residues may influence long range bonding interactions that can influence protein dimerization and DNA binding. Noteworthy is a tyrosine at position 182 that was conserved in 25 strains including Mu50 and Newman, but which is a phenylalanine in NCTC 8325. Recent studies have reported that substitution of the equivalent tyrosine in *Ec*BirA (Y178) disrupts an electrostatic network of binding interactions that alters protein dimerization^18,19^. As the Mu50 sequence employed here is the most highly representative homologue amongst *S. aureus*, this sequence should be considered the prototypical *Sa*BirA in future experiments.

## Discussion

In certain bacteria BirA is a bifunctional protein that is both a biotin protein ligase and transcriptional repressor of biotin biosynthesis. The biosynthesis of biotin is metabolically costly, requiring the activity of at least four gene products and 20 ATP equivalents per biotin molecule^36^. Therefore, the activity of BirA allows cells to find the necessary balance between cellular demand, supply from the environment and *de novo* synthesis. Here we study the BirA protein from two clinically important bacteria, namely *E. coli* and *S. aureus*. *E. coli* is considered the prototypical bacteria, and its BirA protein has been the subject of many biochemical, structural and genetic studies. The *S. aureus* homologue has not been investigated in as much detail. The two bacteria colonize different micro niches where the availability of environmental biotin is distinctive. *E. coli* is a part of the intestinal microflora which contributes to the synthesis and release of substantial amounts of biotin. Recent mouse studies estimate the concentration of biotin in the ileum to be 450–700 nM^37^. Conversely, *S. aureus* possess the extraordinary ability to colonise a wide range of niche microenvironments and is responsible for various disorders affecting the skin, respiratory organs, soft tissues, bones and joints. The bioavailability of biotin at these sites is lower than in the intestine. Accordingly, we propose that *S. aureus* BirA has evolved unique properties that allow the bacteria to adapt to low biotin environments. In this study, we performed detailed *in vivo* and *in vitro* analyses to obtain new insights into the transcription regulation mechanisms of *Sa*BirA. We utilized the *Sa*BirA from the Mu50 methicillin and vancomycin resistant strain of *S. aureus*^38^. Our bioinformatics analysis has demonstrated this particular variant of *Sa*BirA represents the most conserved *Sa*BirA sequence among *S. aureus* strains. Furthermore, Mu50 *Sa*BirA has been the target for the discovery of new classes of antibiotics designed to treat MRSA infections^23,39–41^. We also performed our study alongside the well-characterized BirA from *E. coli*, in order to highlight the differences in dimerization and DNA-binding mechanisms between the two homologues.

Previous studies performed on Mu50 *Sa*BirA revealed weak dimerization of the apo-form of this protein (*K*_D_^2-1^ = 29 ± 0.2 μM)^29^ suggesting that in its apo-state, this protein is likely to be monomeric within the intracellular environment. Hence, we proposed that *Sa*BirA-F123G, as a dimerization-deficient mutant in solution, would mimic the oligomeric state of this apo-*Sa*BirA *in vivo*. Whilst the analogous *E. coli* mutant was clearly devoid of DNA-binding activity *in vivo*, as reported in the literature^31^ and seen here, our results clearly demonstrated *Sa*BirA-F123G functioned as an effective transcriptional repressor for all three *S. aureus* target promoters *in vivo*. Furthermore, even at environmental biotin concentrations as low as 1 nM, wild-type *Sa*BirA and *Sa*BirA-F123G demonstrated some repression compared to the no repressor control. This phenomenon was in contrast to *Ec*BirA. This suggests that *Sa*BirA can either respond to lower concentrations of environmental biotin (unlikely since *K*_M_ values for biotin are similar (0.3 ± 0.1 μM for *Ec*BirA^42^ v 1.01 ± 0.16 μM for *Sa*BirA^23^), or that *Sa*BirA partially occupies the DNA even when biotin is limiting, and therefore, the protein is in the apo-state.

*In vitro* analysis supported the contention that the intrinsically monomeric *Sa*BirA-F123G or wild-type apo-*Sa*BirA can bind DNA, enabling *in vivo* repression. The Mu50 strain wild-type *Sa*BirA used here was unable to dimerize in solution at a protein concentration of 10 µM in its apo-form, as indicated by native nESI-MS. This finding agrees with our previous work which has demonstrated the apo-protein to be monomeric by small angle X-ray scattering assay^29^ and crystallises as a single subunit^24^. It also agrees with recent work conducted on *Sa*BirA from the Newman strain reported by Wang and Beckett^25^ where analytical ultracentrifugation (AUC) failed to detect homodimerization. This is in contrast to our previous AUC datum where dimerisation of apo-*Sa*BirA from Mu50 was observed^29^. It should be noted that different protein sequences were employed between these two studies (discussed further below). Additionally, this may have been the result of contaminating co-repressor, biotinyl-5′-AMP, that was not detected by the Streptavidin blot method used to assay the purified protein^27^. Here, a superior technique of native nESI-MS has been utilized to unambiguously detect not only the oligomeric state of the protein, but to simultaneously determine whether these species are truly devoid of bound ligands. This is the first report where native mass spectrometry has been employed to study BirA. No dimeric form of *Sa*BirA-F123G was observed in either the apo or holo forms in solution, supporting its characterization as a monomeric mutant.

Furthermore, our results suggest dimerization of apo-*Sa*BirA and *Sa*BirA-F123G is mediated by DNA binding, as native nESI-MS detected species with molecular weights equivalent to two monomers of *Sa*BirA in complex with the *SabioY* recognition sequence oligonucleotide. Likewise, nESI-MS revealed wild-type and *Sa*BirA-F123G bound a DNA probe containing only a single half site in both the apo and holo-states but with different stoichiometry equivalents. Whilst the apo-proteins only bind as one subunit, biotinyl-5′-AMP allows the dimerization of wild-type *Sa*BirA prior to DNA binding but also allowed *Sa*BirA-F123G to dimerise on the DNA, despite not detectably dimerising in solution. The binding of *Sa*BirA-F123G to DNA agrees with previous studies on apo-*Sa*BirA where the *K*_D_ for DNA binding was 6-fold lower compared to holo-*Sa*BirA as measured by EMSA^29^, and 60-fold lower as measured by fluorescence anisotropy^27^.

**Figure 7:**
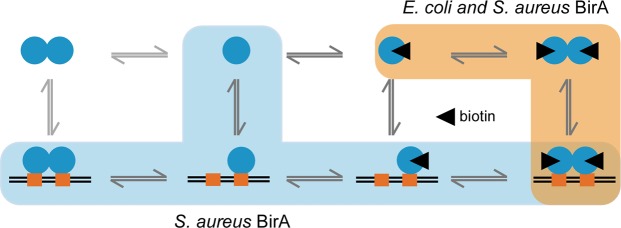
Proposed DNA binding pathway of *Sa*BirA. In biotin-limiting conditions, BirA cannot dimerise in solution. However, *Sa*BirA monomers (blue ovals) can bind to individual half sites (orange boxes), with the interaction presumably stabilized by interaction between the monomers on the DNA. Under high biotin conditions (biotin is indicated by a black triangle), *Sa*BirA follows the same binding mechanism as holo-*Ec*BirA, where a pre-formed dimer binds to DNA (light orange shading).

By combining our *in vitro* and *in vivo* data, we propose two possible pathways for *Sa*BirA to assemble on DNA, as illustrated in Fig. 7. In high biotin conditions, protein dimerization occurs in solution and precedes DNA binding, as has been well documented for *Ec*BirA^5,7^. The pre-formed homodimer orients the two helix-turn-helix motifs present in the N-terminus of the protein such that they can simultaneously occupy both half sites on the target operator. Here we present evidence that binding to DNA can also proceed via the sequential assembly of two monomers onto the DNA, a pathway which is much less preferred for *Ec*BirA. This alternative pathway is likely be the basis for the lower biotin threshold required to initiate the transcriptional repression observed with *Sa*BirA compared to *Ec*BirA, as highlighted in a previous study^26^ and seen with the partial occupation of *Sa*BirA on DNA causing repression at low biotin concentrations (1 nM) in the *in vivo* reporter assay^26^. Whilst the DNA mediated dimerization pathway has been observed with the *Sa*BirA from the Mu50 strain, which we have identified here as the prototypical example in *S. aureus*, it remains to be seen whether BirA from other species behave in a similar manner. The findings from this study may provide new insights into how biotin-regulated gene expression occurs in *S. aureus* allowing it to adapt to the low biotin environments it colonises.

Our bioinformatics analysis has identified multiple polymorphisms amongst *S. aureus* strains which may give rise to BirA variants with different homo-dimerization and DNA binding activities. Mutagenesis studies that target specific amino acids in *Ec*BirA, together with structural biology and biophysical assays, are helping to understand mechanisms of allosteric communication between distant sites in the protein and the impact upon their function^18,21,35^. Noteworthy is mutation of tyrosine 178 to cysteine (equivalent to Y182 in *S. aureus* Mu50, and F182 in NCTC 8325) that results in a super-repressor phenotype in whole cell reporter assays of transcription^18^. This amino acid does not reside directly in the dimer interface but, instead, contributes to complex network of electrostatic interactions that are proposed to influence the structural alignment of the central (ligand-binding) and N-terminal (DNA-binding) domains of BirA that effect homo-dimerization and DNA binding^19^. Examination of available crystal structures of the Mu50 *Sa*BirA^23,24,29^ likewise revealed that Y182 contributes to a network of electrostatic interactions involving K176 (that resides in a conserved KWPND motif in the active site) and D322 that resides in the DNA binding domain. Our analysis of genomic information also identified two differences in the BirA proteins from Mu50 and Newman at positions 247 and 272. Mutational studies, such as those performed to characterise the *Ec*BirA mutants^18,19^, are required to probe the structure-function relationships in more detail. We propose that the *Sa*BirA variants identified here present a useful system with which to further probe long distance modulation of protein allostery.

## Methods

### Materials

*E. coli* strain JD26186 (*bioC*::Kan), based on host strain KP7600, is a transposon-disrupted bioC biotin auxotroph, and was obtained from the National BioResource Project (NIG, Japan). Bacteria were cultured at 37 °C with vigorous shaking in Luria Bertani media containing the appropriate antibiotic. Plasmid extractions were performed using the Plasmid QIAprep Spin Miniprep Kit (Qiagen) and genomic extractions were performed using the Wizard® Genomic DNA purification kit (Promega). All molecular biology enzymes (DNA polymerase, ligases and restriction enzymes) and buffers were supplied by New England Biolabs. Oligonucleotides, purchased from Geneworks Ptd Ltd, are shown in Supporting Table S1.

### Nucleic acid manipulations

All plasmids employed in this study are shown in Supporting Table S2. Site directed mutagenesis (Quikchange Site Directed Mutagenesis kit, Stratagene), was performed upon plasmid pGEM-*Ec*BirA-H6^29^ using oligonucleotides B460 and B461, yielding pGEM-*Ec*BirA-R119W-H6. The *EcbirA-R119W-H6* coding region was subsequently cloned into either pET16b (Novagen) for recombinant protein expression, or the integration vector pIT4_TL_152002^43^, yielding pIT4_TL_*Ec*BirA-R119W-H6. Similarly, the *SabirA-F123G-H6* coding region was excised from pGEM-*Sa*BirA-F123G-H6^29^ and ligated into pIT4_TL_152002, yielding pIT4_TL_*Sa*BirA-F123G-H6.

### Generation of bacterial strains for *in vivo* reporter assays

All strains of *E. coli* employed in this study are shown in Supporting Table S3. β-galactosidase reporter assays were performed on *E. coli* reporter strains generated in^26^. Both the β-galactosidase reporters and the BirA expression modules were site-specifically integrated in the chromosome in single copy (Fig. 1A). BirA binding sequence was cloned into pLacZ_SHTrim plasmid upstream of the β-galactosidase reporter gene. BirA expression (*Ec*BirA, *Sa*BirA and their dimerization defective mutants *Ec*BirA-R119W and *Sa*BirA-F123G) was under control of the placUV5 promoter. These were constructed using the methods outlined in^26,44^. The β-galactosidase reporter assays were conducted as described^43,45^.

### Protein methods

Recombinant expression of all BirA proteins employed in this study, as well as their purification by immobilized metal ion affinity chromatography (IMAC), were performed essentially as previously described^29,34^. However, the following modifications were included to ensure removal of biotinyl-5′-AMP from the protein preparations. Prior to IMAC purification, the cell lysate was incubated at 4 °C for a minimum of 1 hour with 10 μL 50% slurry Streptavidin-sepharose High Performance (GE Healthcare Life Sciences) per millilitre of lysate. This was centrifuged at 4000 rpm, 4 °C for 10 minutes, to remove the resin. The cleared lysate was then incubated for 1 hour at 37 °C with 6 mg biotin-accepting substrate GST-*Sa*PC90 per 10 ml of lysate prior to IMAC. Purified proteins were dialyzed overnight in 4 L of storage buffer (50 mM Tris pH 8.0, 100 mM KCl, 1 mM DTT, 5% (v/v) glycerol) and stored at −80 °C until required. Protein SDS-PAGE was performed using NuPage^TM^ Bis-Tris 4–12% gels (Invitrogen).

### Native nano-electrospray ionisation-mass spectrometry (nESI-MS)

Purified BirA proteins were buffer exchanged into 200 mM ammonium acetate pH 6.85 (Sigma) using Amicon Ultra-0.5 MWCO 10,000 centrifugal filter units (Merck Millipore). Holo-enzyme samples (at least 100 µM, except for *Ec*BirA-R119W that was 30 µM) were prepared by pre-incubating the apo-proteins with 500 µM biotin, 1 mM ATP and 1 mM MgCl_2_ on ice for at least 1 hour prior to buffer exchange. HPLC-purified and annealed, double stranded oligonucleotides containing the operator sequences of interest were purchased from Integrated DNA Technology (USA). These were desalted into 200 mM ammonium acetate using Illustra MicroSpin G-25 columns (GE Healthcare). Oligonucleotide concentrations were quantified using a Nanodrop spectrophotometer (Thermo Fisher Scientific). Proteins were quantified following buffer exchange via Bradford assay, and then diluted to 10 µM in 200 mM ammonium acetate for analysis by nESI-MS. Protein:DNA complexes were analyzed by nESI-MS after diluting the oligonucleotide and protein in 200 mM ammonium acetate pH 6.9 to 5 µM and 10 µM respectively, and incubating at 4 °C for at least 1 hour prior to MS analysis.

Mass spectrometry (MS) measurements were performed on a Synapt HDMS system (Waters, UK) with the sample introduced by nano-electrospray ionization in the positive ion MS mode from platinum-coated borosilicate capillaries prepared in-house. Instrument parameters were optimized to remove adducts while preserving non-covalent interactions, and were as follows; capillary voltage, 1.5 kV; cone voltage, 60 V; trap collision energy, 20 V; transfer collision energy, 15 V; source temperature, 50 °C; backing pressure, 3.95 mbar. Data analysis was performed using manual peak finding in MassLynx software (version 4.1).

### Electrophoretic mobility shift assay (EMSA)

Binding reactions were performed at room temperature for 30 minutes using EMSA buffer (50 mM Tris pH 8.0, 50 mM NaCl, 0.1 mM biotin, 1 mM ATP, 1 mM MgCl_2_ and 10% (v/v) glycerol) together with 10 nM of the double stranded oligonucleotide (HPLC-purified as utilized for nESI-MS) and varying concentrations of BirA. Fractionation of the samples was performed using 4–12% TBE polyacrylamide gels (Life Technologies) run at 100 volts (constant) for 45 minutes and stained in GelRed (Biotium) solution for 5 minutes. After washing five times in distilled water, the gels were imaged using a ChemiDoc imager (Bio-Rad). Each EMSA experiment was performed in triplicate.

### *S. aureus* BirA multiple sequence alignment

BirA sequences of *S. aureus* were compared as follows: the protein cluster PCLA_885364 (accessed on 15/5/17) containing 71 entries (which encompasses all non-redundant proteins identified as biotin-acetyl-CoA-carboxylase ligase in the Genus Staphylococcus) was obtained from NCBI^46,47^ and further filtered for the clade *Staphylococcus aureus* 19988 to make a list of 26 unique protein sequences. These sequences were then aligned using Clustal Omega^48^. The alignment was then compared using UGENE^49^ and the position of the polymorphisms were mapped onto the *Sa*BirA structure (PDB accession 4DQ2^23^) using UCSF Chimera software (version 1.12). The *Ec*BirA sequence for comparison was obtained from the PDB accession 2EWN^6^.

## Supplementary information

Supplementary Information
