# Supplementary material for: Native mass spectrometry identifies an alternative DNA-binding pathway for BirA from Staphylococcus aureus
Source: Sci Rep. 2019 Feb 26;9:2767. doi: 10.1038/s41598-019-39398-6 (PMC6391492; doi:10.1038/s41598-019-39398-6)
Supplement: Supplementary file 1 — Supplementary Information [file 41598_2019_39398_MOESM1_ESM.pdf]

Supporting Information

Native mass spectrometry identifies an alternative DNA-binding pathway for BirA from *Staphylococcus aureus*

Jiulia Satiaputra[1#a], Louise M. Sternicki[1#], Andrew J. Hayes[1b], Tara. L. Pukala[2], Grant W. Booker[1], Keith E. Shearwin[1] and Steven W. Polyak[1*c]

[1] School of Biological Sciences, University of Adelaide, South Australia 5005, Australia;

[2] School of Physical Sciences, University of Adelaide, South Australia 5005, Australia

Present Address: [a] Harry Perkins Institute of Medical Research, Western Australia, 6008, Australia. [b] Faculty of Health and Medical Sciences, Adelaide, South Australia, 5005, Australia. [c] School of Pharmacy and Medical Sciences, University of South Australia, South Australia 5001.

* To whom correspondence should be addressed. Tel: +61 8 8313 4062; Email: steven.polyak@adelaide.edu.au

#J.S. and L.M.S. should be considered joint first author.

Supporting Figures

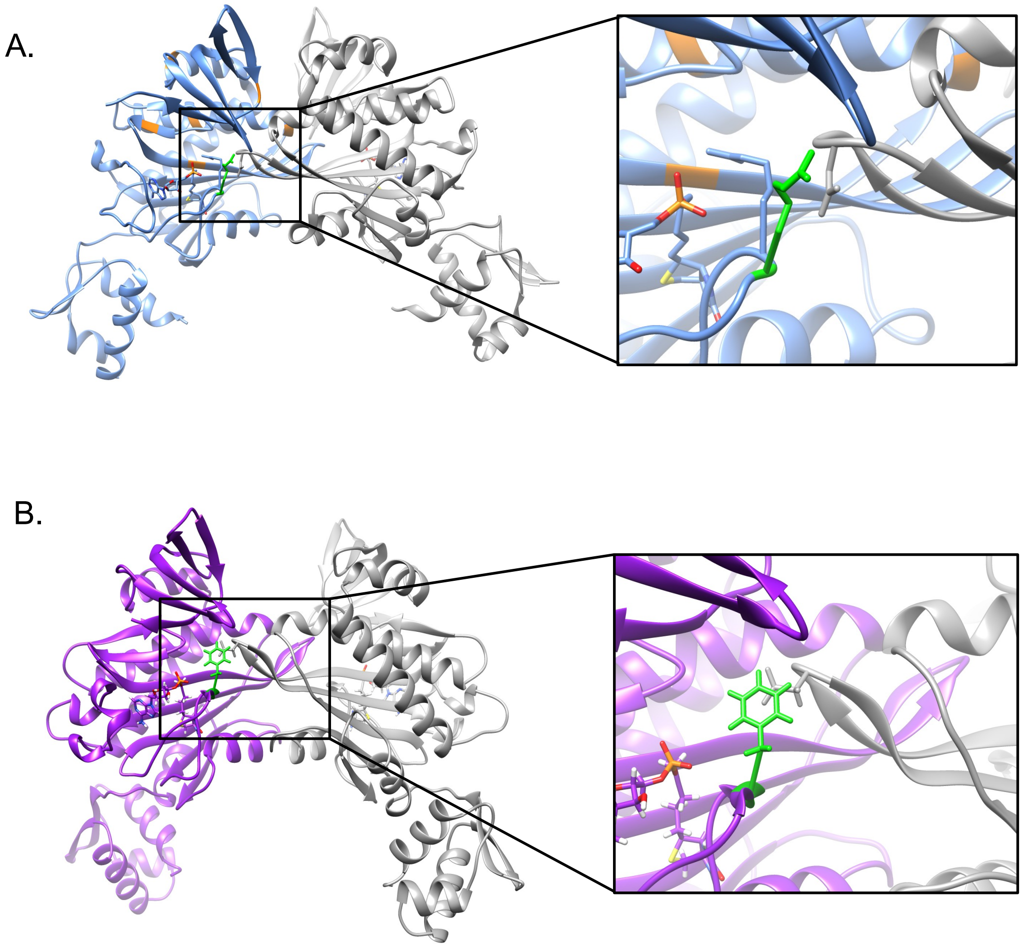

Supporting Figure S1. The dimeric structures of (A) *Ec*BirA and (B) *Sa*BirA reveal the equivalent residues are mutated to make the dimerization-impaired mutants *Ec*BirA-R119W and *Sa*BirA-F123G, respectively.

*Ec*BirA (PDB 2EWN) [1] is shown with one monomer in blue and the other in grey, whilst *Sa*BirA (PDB 4DQ2) [2] has one monomer shown in purple and the other in grey. The residues that are mutated to produce the monomeric mutants are shown in green, whilst the residues with which they interact in the partner subunit are in grey. Both *Ec*BirA and *Sa*BirA have the co-repressor (and reaction intermediate) analogue, biotinol-5′-AMP, bound.

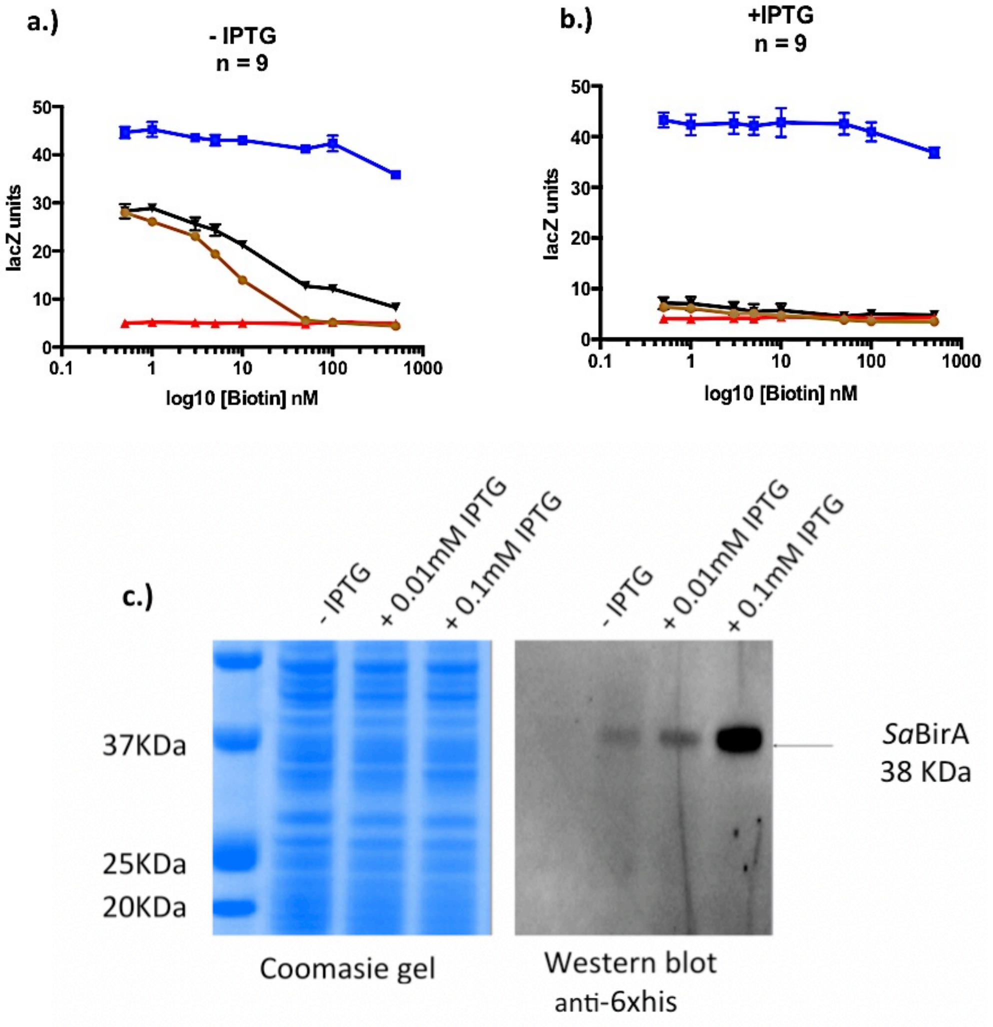

Supporting Figure S2. Repressor activity in the presence and absence of IPTG.

In vivo repressor activity of *Sa*BirA (blue curves) and *Sa*BirA F123G (brown curves) in either a) the absence of IPTG or (b) 0.01mM IPTG. The blue curves represent no-repressor controls and red curves represent no-promoter controls. β-galactosidase activity was measured in response to varying concentrations of biotin in the growth media. Error bars denote S.E.M. of n = 9. (c.) IPTG inducible expression of *Sa*BirA-H6 was detected by Western blot probed with anti-H6 antibody (right panel). A Coomassie blue stained gel was included as a control for protein loading (left panel).

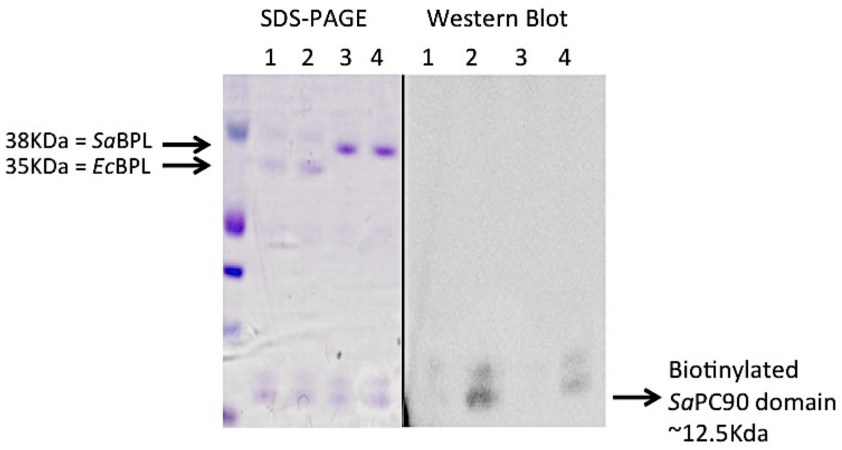

Supporting Figure S3. Biotinyl transferase assay.

Streptavidin-blot analysis was performed upon the products of an *in vitro* biotinylation assay to confirm biotinyl-5′-AMP did not co-purify with apo-*SaBirA* or apo-*Ec*BPL, as previously described [3]. The assay used SaPC90 as the biotin-accepting protein substrate. Reactions containing either (1) apo-*Ec*BPL, (2) holo-*Ec*BPL, (3) apo-*SaBirA* or (4) holo-*SaBirA* were investigated. The absence of bands on the Streptavidin blot in lanes 1 and 3 suggested biotinyl-5′-AMP had not been co-purified with the BirAs. The corresponding SDS-PAGE analysis is presented as a loading control.

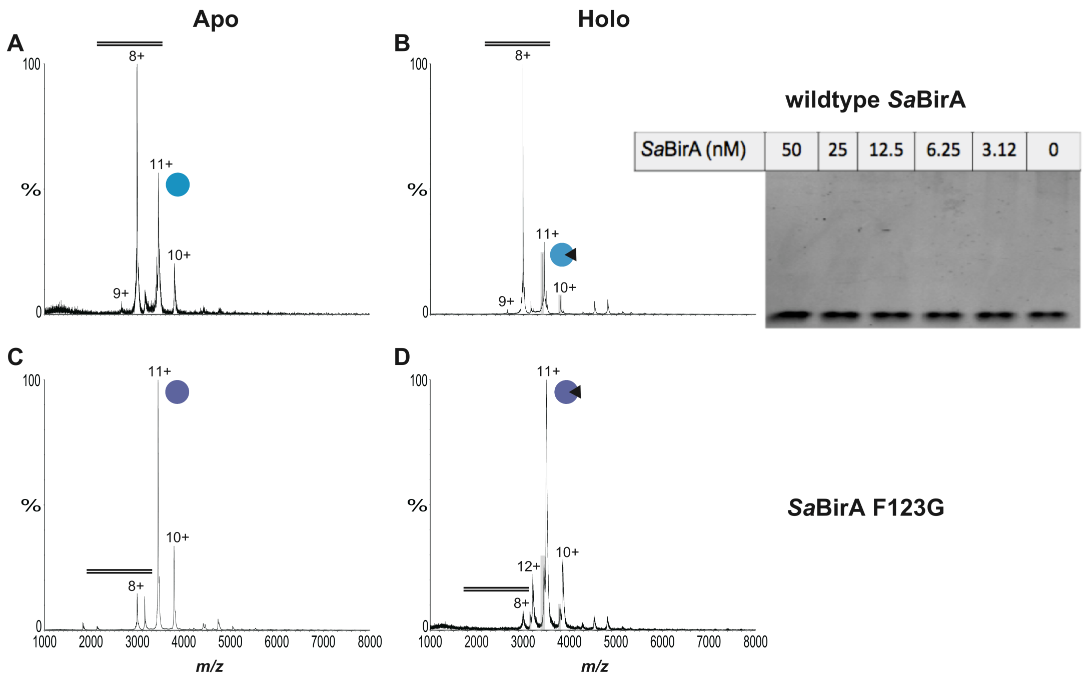

5′-GCCTTACACAGACGATTATTAATTATAAAAAGCAGACACCGGAT-3′

Supporting Figure S4. Control for non-specific binding between *Sa*BirA and a mutated *SabioO* oligonucleotide containing no BirA binding sites.

A protein:DNA complex was not detected by native nESI-MS using a mutated oligonucleotide and (A) apo-*Sa*BirA, (B) holo-*Sa*BirA, (C) apo-*Sa*BirA-F123G or (D) holo-*Sa*BirA-F123G. The DNA sequence is shown with the mutated half site sequences highlighted in red. Similarly, EMSA analysis failed to detect protein binding (insert). Within the MS spectra, double black lines represent the oligonucleotide, light blue spheres signify *Sa*BirA and dark purples are *Sa*BirA-F123G. Black triangles signify the presence of biotinyl-5′-AMP and, therefore, holo-protein. Measured masses of all the products are shown in Supporting Table S6.

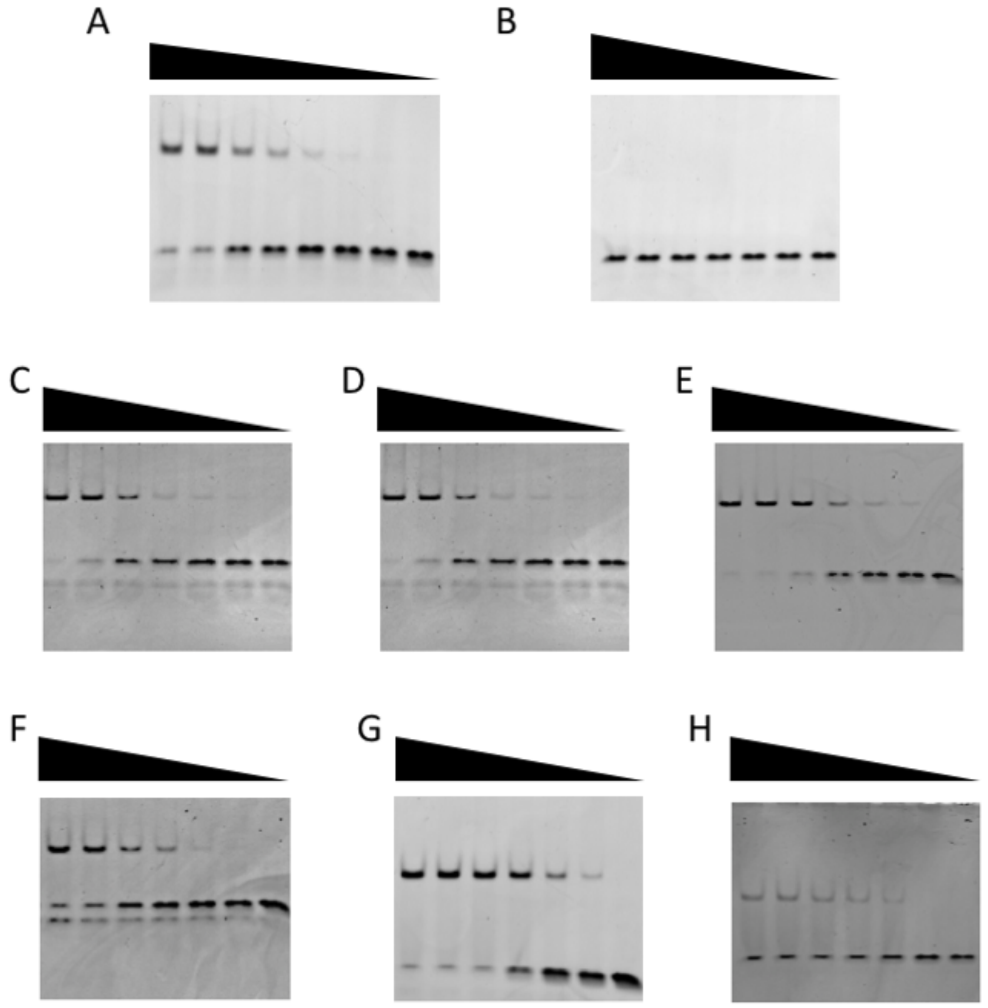

Supporting Figure S5. Full length blots from cropped blots in Figure 4.

EMSA of the interaction between *EcbioO* and (A) *Ec*BirA or (B) *Ec*BirA-R119W. Also shown is *Sa*BirA binding to (C) *SabioO,* (D) *bioY* or (E) *yhfS-yhfT* and *Sa*BirA-F123G binding to (F) *SabioO,* (G) *bioY* and (E) *yhfS-yhfT.* Concentrations of enzyme used in the binding reactions are shown in Figure 4.

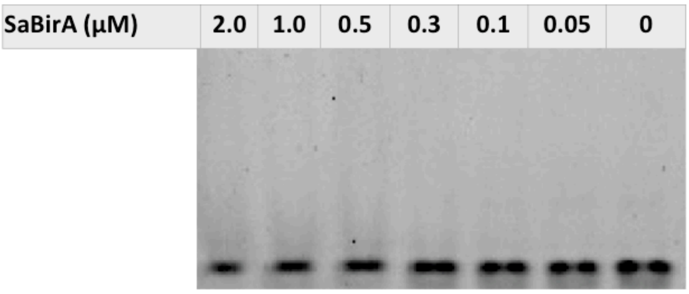

AATGTAAAC
5′-GCCTTACACAGACGATTATTAATTATAAAAGTTTACATTCGGAT-3′

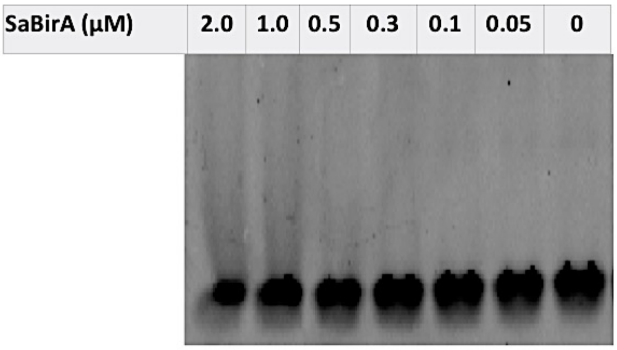

GTTTACATT
5′-GCCTTAAATGTAAACTTATTAATTATAAAAAGCAGACACCGGAT-3′

Supporting Figure S6. EMSA analysis of *Sa*BirA binding to a *SabioO* oligonucleotide containing a single BirA half site.

EMSA analysis was performed on double stranded *SabioO* oligonucleotides containing (top panel) the first half-site mutated and (lower panel) the second half-site mutated. The oligonucleotides are shown below the EMSA with the mutated sequence in red, the wild-type sequence is underlined and the half sites highlighted in yellow. For both oligonucleotides, no DNA binding was observed for *Sa*BirA (up to a concentration of 2 µM).

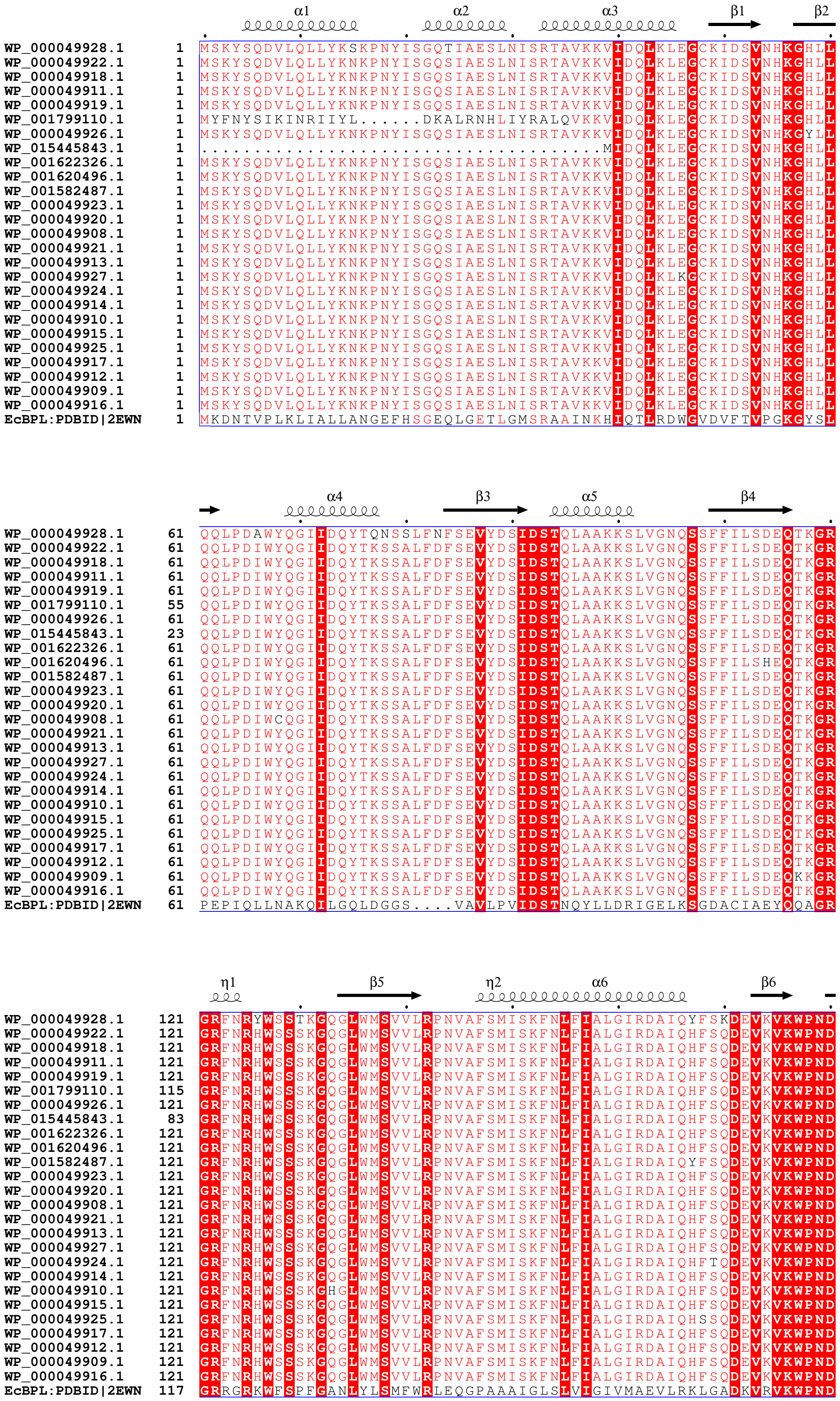

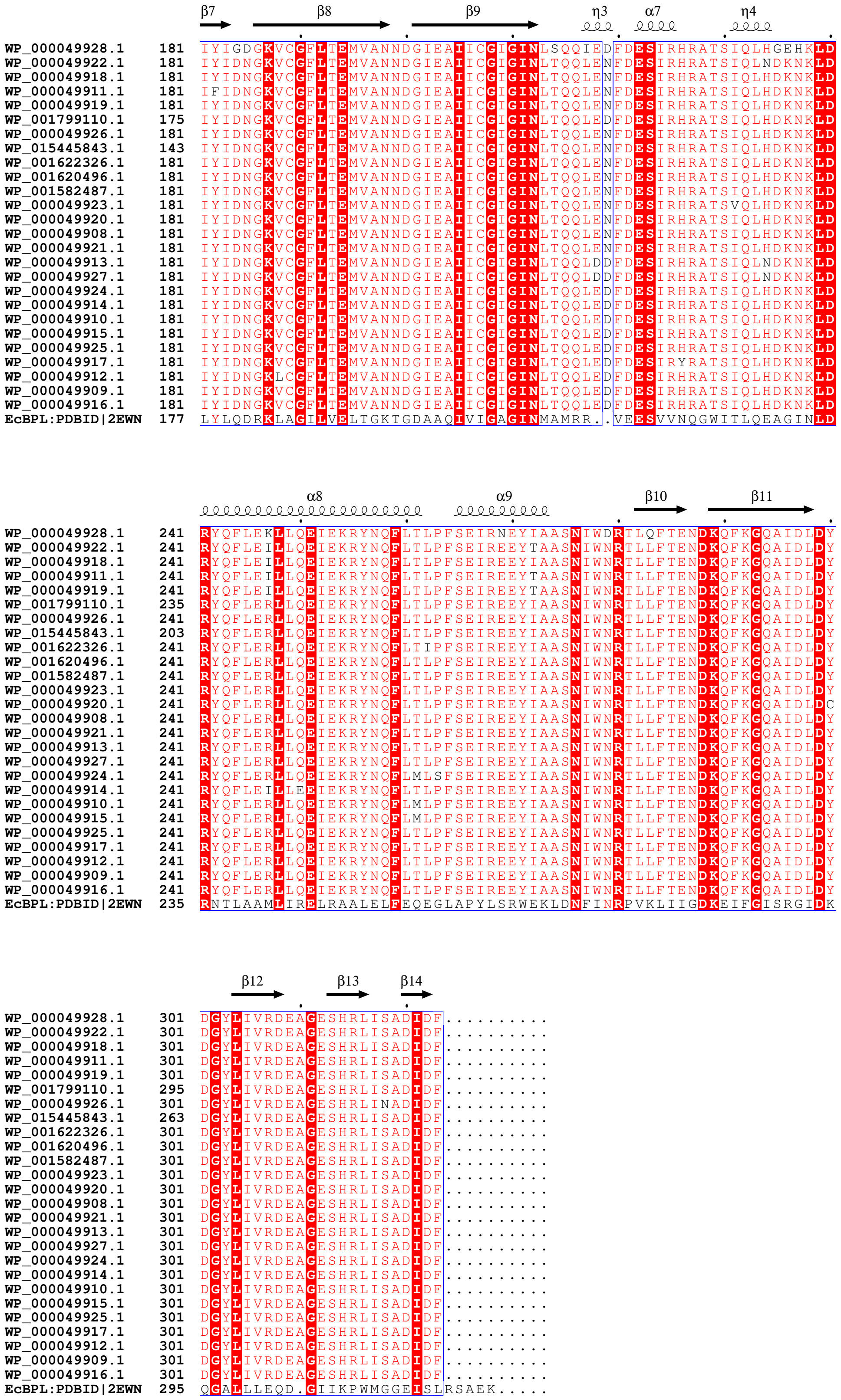

Supporting Figure S7. Alignment of 26 non-redundant *Sa*BirA sequences and the *E. coli* homologue. Invariant residues are boxed in red, whilst highly conserved residues are in red text. Black text indicates the presence of a polymorphism, or divergence from the *Ec*BirA sequence (bottom sequence in the alignment).

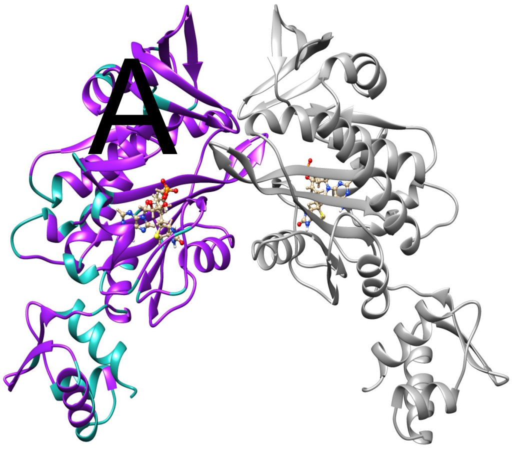

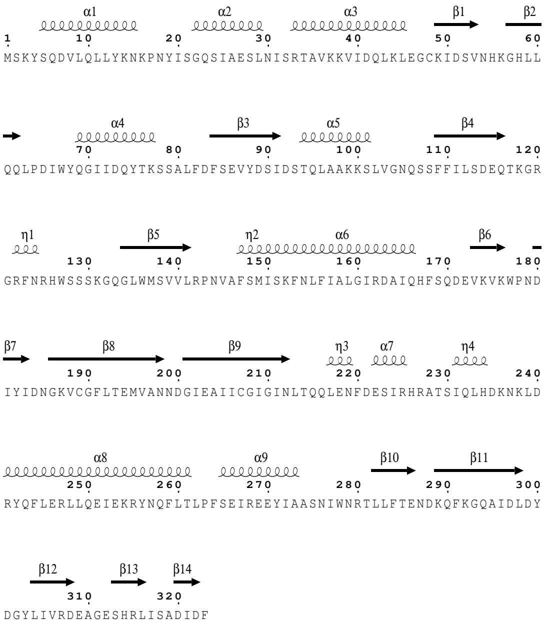

Position	Consensus	Strain	Polymorphism	Domain localisation	Structural Element	Possible effect
2	S	WP_001799110.1	Y	N-terminal DNA binding domain		
3	K	WP_001799110.1	F	N-terminal DNA binding domain		
4	Y	WP_001799110.1	N	N-terminal DNA binding domain		
5	S	WP_001799110.1	Y	N-terminal DNA binding domain	α-helix 1	
6	Q	WP_001799110.1	S	N-terminal DNA binding domain	α-helix 1	
7	D	WP_001799110.1	I	N-terminal DNA binding domain	α-helix 1	
8	V	WP_001799110.1	K	N-terminal DNA binding domain	α-helix 1	
9	L	WP_001799110.1	I	N-terminal DNA binding domain	α-helix 1	
10	Q	WP_001799110.1	N	N-terminal DNA binding domain	α-helix 1	
11	L	WP_001799110.1	R	N-terminal DNA binding domain	α-helix 1	
12	L	WP_001799110.1	I	N-terminal DNA binding domain	α-helix 1	
13	Y	WP_001799110.1	I	N-terminal DNA binding domain	α-helix 1	
14	K	WP_001799110.1	Y	N-terminal DNA binding domain	α-helix 1	
15	N	WP_000049928.1	S	N-terminal DNA binding domain	α-helix 1	
15	N	WP_001799110.1	L	N-terminal DNA binding domain	α-helix 1	
22	G	WP_001799110.1	D	N-terminal DNA binding domain	α-helix 2	
23	Q	WP_001799110.1	K	N-terminal DNA binding domain	α-helix 2	
24	S	WP_000049928.1	T	N-terminal DNA binding domain	α-helix 2	
24	S	WP_001799110.1	A	N-terminal DNA binding domain	α-helix 2	
25	I	WP_001799110.1	L	N-terminal DNA binding domain	α-helix 2	
26	A	WP_001799110.1	R	N-terminal DNA binding domain	α-helix 2	
27	E	WP_001799110.1	N	N-terminal DNA binding domain	α-helix 2	
28	S	WP_001799110.1	H	N-terminal DNA binding domain	α-helix 2	
30	N	WP_001799110.1	I	N-terminal DNA binding domain		
31	I	WP_001799110.1	Y	N-terminal DNA binding domain		
32	S	WP_001799110.1	R	N-terminal DNA binding domain		
33	R	WP_001799110.1	A	N-terminal DNA binding domain	α-helix 3	
34	T	WP_001799110.1	L	N-terminal DNA binding domain	α-helix 3	
35	A	WP_001799110.1	Q	N-terminal DNA binding domain	α-helix 3	
39	V	WP_015445843.1	M	N-terminal DNA binding domain	α-helix 3	
46	E	WP_000049927.1	K	N-terminal DNA binding domain		
58	H	WP_000049926.1	Y	N-terminal DNA binding domain	β-sheet 2	
66	I	WP_000049928.1	A	N-terminal DNA binding domain		

68	Y	WP_000049908.1	C	catalytic domain		
77	K	WP_000049928.1	Q	catalytic domain	α-helix 4	
78	S	WP_000049928.1	N	catalytic domain		
80	A	WP_000049928.1	S	catalytic domain		
83	D	WP_000049928.1	N	catalytic domain		
114	D	WP_001620496.1	H	catalytic domain	β-sheet 4	next residue (115) contacts biotinol-5′-AMP
117	T	WP_000049909.1	K	catalytic domain		next to the biotin-binding loop (residues 118-128)
126	H	WP_000049928.1	Y	catalytic domain		in biotin-binding loop, contacts biotinol-5′-AMP
130	S	WP_000049928.1	T	catalytic domain		near the biotin-binding loop (residues 118-128)
133	Q	WP_000049910.1	H	catalytic domain		
167	H	WP_000049928.1	Y	catalytic domain		
167	H	WP_001582487.1	Y	catalytic domain		
168	F	WP_000049925.1	S	catalytic domain		
169	S	WP_000049924.1	T	catalytic domain		
170	Q	WP_000049928.1	K	catalytic domain		
182	Y	WP_000049911.1	F	catalytic domain	β-sheet 7	
184	D	WP_000049928.1	G	catalytic domain		
185	N	WP_000049928.1	D	catalytic domain		
188	V	WP_000049912.1	L	catalytic domain	β-sheet 8	previous residue (187) contacts biotinol-5′-AMP
214	T	WP_000049928.1	S	catalytic domain		next residue (215) contacts biotinol-5′-AMP
217	L	WP_000049928.1	I	catalytic domain	3_{10}-helix 3	
218	E	WP_000049913.1	D	catalytic domain	3_{10}-helix 3	
218	D	WP_000049927.1	D	catalytic domain	3_{10}-helix 3	
219	D/N	All strains	D/N	catalytic domain	3_{10}-helix 3	next residue (220) contacts biotinol-5'-AMP
226	H	WP_000049917.1	Y	catalytic domain		next residues (227-229) contact biotinol-5'-AMP
231	I	WP_000049923.1	V	catalytic domain	3_{10}-helix 4	
234	H	WP_000049922.1	N	catalytic domain	3_{10}-helix 4	
234	H	WP_000049913.1	N	catalytic domain	3_{10}-helix 4	
234	H	WP_000049927.1	N	catalytic domain	3_{10}-helix 4	
235	D	WP_000049928.1	G	catalytic domain		
236	K	WP_000049928.1	E	catalytic domain		
237	N	WP_000049928.1	H	catalytic domain		
247	R	WP_000049928.1	K	catalytic domain	α-helix 8	
247	R	WP_000049922.1	I	catalytic domain	α-helix 8	
247	R	WP_000049918.1	I	catalytic domain	α-helix 8	
247	R	WP_000049911.1	I	catalytic domain	α-helix 8	
247	R	WP_000049919.1	I	catalytic domain	α-helix 8	
247	R	WP_000049914.1	I	catalytic domain	α-helix 8	
250	Q	WP_000049914.1	E	catalytic domain	α-helix 8	
261	T	WP_000049924.1	M	catalytic domain	α-helix 8	
261	T	WP_000049910.1	M	catalytic domain	α-helix 8	
261	T	WP_000049915.1	M	catalytic domain	α-helix 8	
262	L	WP_001622326.1	I	catalytic domain		
263	P	WP_000049924.1	S	catalytic domain		
269	E	WP_000049928.1	N	C-terminal cap	α-helix 9	
272	I	WP_000049922.1	T	C-terminal cap	α-helix 9	
272	I	WP_000049911.1	T	C-terminal cap	α-helix 9	
272	I	WP_000049919.1	T	C-terminal cap	α-helix 9	
279	N	WP_000049928.1	D	C-terminal cap		
283	L	WP_000049928.1	Q	C-terminal cap	β-sheet 10	
300	Y	WP_000049920.1	C	C-terminal cap		
318	S	WP_000049926.1	N	C-terminal cap		involved in the dimer interface

Supporting Figure S8. Polymorphisms present in the various *S. aureus* strains, and their potential impact due to their structural localization.

(A) Location of polymorphisms (cyan) is shown in the ribbon diagram of *Sa*BirA detected from the sequence alignment (Supporting Figure S7). (B) Sequence of the Mu50 strain *Sa*BirA with secondary structures from the crystal structure annotated. (C) Table of all the polymorphisms within the 26 non-redundant *Sa*BirA sequences identified from the multiple sequence alignment (Supporting Figure S7). The position of the mutations in the crystal structure is annotated.

Supporting Table S1: List of oligonucleotides employed in this study.

Oligo name	Sequence 5'-3'	Description
Cloning oligonucleotides		
B386	GACTAAAATGTTGAATCGCATTCTTATCCCTAAATCAATAA ATAAATTAAATTTAGATATCATTGAGAATGC	Sequencing primer to sequence pTac-SaBirA in integration plasmid pIT4_TL152002
HK022-P1	GGAATCAATGCCTGAGTG	attp-HK022 PCR screening primer [4]
HK022-P2	ACTTAACGGCTGACATGG	attp-HK022 PCR screening primer [4]
HK022-P3	ACGAGTATCGAGATGGCA	attp-HK022 PCR screening primer [4]
HK022-P4	GGCATCAACAGCACATTC	attp-HK022 PCR screening primer [4]
Lambda P1	GGCATCACGGC AATATAC	attp-λ PCR screening primer [4]
Lambda P2	ACTTAACGGCTGACATGG	attp-λ PCR screening primer [4]
Lambda P3	GGGAATTAATTCTTGAAGACG	attp-λ PCR screening primer [4]
Lambda P4	TCTGGTCTGGTAG CAATG	attp-λ PCR screening primer [4]
B460_R119W_F	GGCCGTGGTCGCTGGGGTCGGAAATGG	Forward mutagenesis primer for EcBirA-R119W
B461_R119W_R	CCATTTCCGACCCCAGCGACCACGGCC	Reverse mutagenesis primer for EcBirA-R119W
B479	GACTCATCATGAAGGATAACACCGTGCCAC	Forward primer to clone EcBirA-R119W into integration plasmid pIT4_TL 152002
B320	ACTAGTGATAAGCTTAATGATGATGATGATGATGTCC	Reverse primer to clone EcBirA-R119W into integration plasmid pIT4_TL_152002
EMSA and native MS oligonucleotides		
DS-SaBioO oligo 1	CCTTAAATGTAAACTTTTATAATTAATAAGTTTACATTTAAG	Top strand oligo containing SabioO wildtype sequence
DS-SaBioO oligo 2	CCTTAAATGTAAACTTATTAATTATAAAAGTTTACATTTAA GG	Bottom strand oligo containing SabioO wildtype sequence
DS-SabioY oligo 1	AACTTATTGTAAACTTTTCATTTCTTAAAGTTTACAATGGTG CT	Top strand oligo containing SabioY wildtype sequence

DS-SabioY oligo 2	AGCACCATTGTAAACTTTAAGAAATGAAAAGTTTACAATAA GTT	Bottom strand oligo containing SabioY wildtype sequence
DS-yHFS-T oligo 1	TTATATAATGTTAACAAGATGTATTTTAAAGTTTACATTGA GTGA	Top strand oligo containing yHFS-T wildtype sequence
DS-yHFS-T oligo 2	TCACTCAATGTAAACTTTAAAATACATCTTGTTAACATTAT ATAA	Bottom strand oligo containing yHFS-T wildtype
DS-HS1m oligo 1	TTACACAGACGATTATTAATTATAAAAGTTTACATTCG	Top strand oligo containing mutated sequence of the first half-site of *SaBirA* recognition sequence for SabioO
DS-HS1m oligo 2	CGAATGTAAACTTTTATAATTAATAATCGTCTGTGTAA	Bottom strand oligo containing mutated sequence of the first half-site of SaBirA recognition sequence for SabioO
DS-HS2m oligo 1	TTAAATGTAAACTTATTAATTATAAAAAGCAGACACCG	Top strand oligo containing mutated sequence of the second half-site of SaBirA recognition sequence for SabioO
DS-HS2m oligo 2	CGGTGTCTGCTTTTTATAATTAATAAGTTTACATTTAA	Bottom strand oligo containing mutated sequence of the second half-site of SaBirA recognition sequence for SabioO
DS-HSm oligo 1	TTACACAGACGATTATTAATTATAAAAAGCAGACACCG	Top strand oligo containing mutated sequence of both half-site of SaBirA recognition sequence for SabioO
DS-HSm oligo 2	CGGTGTCTGCTTTTTATAATTAATAATCGTCTGTGTAA	Bottom strand oligo containing mutated sequence of both half-site of SaBirA recognition sequence for SabioO

Supporting Table S2. Plasmids employed in this study.

Plasmid	Description	Reference
pGEMT-EcBirA-H6	pGEMT plasmid containing EcBirA with 6x his-tag	[3]
pIT3_CLlacZ_Trim	Chromosomal integration plasmid (λ-attP, Cm^R, R6Kγ ori, lacZ)	[5]
pIT3-SH-152002	Chromosomal integration plasmid (HK022-attP, $Spec^R$, R6Kγ ori, ccdB, pUC ori)	Shearwin lab, Adelaide University
placZ_SH_Trim	Chromosomal integration plasmid (HK022-attP, $Spec^R$, R6Kγ ori, lacZ)	this study
pIT4_TL_152002	Chromosomal integration plasmid (λ-attP, Tc^R, R6Kγ ori, ccdB, pUC ori)	[4]
pGEMT-EcBirA-R119W-H6	pGEMT plasmid containing EcBirA-R119W-H6	this study
pGEMT-SaBirA F123G-H6	pGEMT plasmid containing SaBirA-F123G-H6	[3]
pET16b-EcBirA-R119W-H6	pET16b expression vector containing EcBirA-R119W-H6	this study
pET16b-SaBirA-F123G-H6	pET16b expression vector containing SaBirA-F123G-H6	[3]
pIT4_TL_SaBirA-H6	plac-UV5 fused with SaBirA-H6 sequence cloned into pIT4_TL_152002	[6]
pIT4_TL_SaBirA-F123G-H6	plac-UV5 fused with SaBirA-F123G-H6 sequence cloned into pIT4_TL_152002	this study
pIT4_TL_EcBirA-R119W-H6	plac-UV5 fused with EcBirA-R119W-H6 sequence cloned into pIT4_TL_152002	this study

Supporting Table S3: Bacterial strains employed in this study.

Strain name	Genotype	Description	Source
KP7600	KP7600 (F- lacIQ lacZdeltaM15 galK2 galT22 lambda- in (rrnD-rrnE)1	W3110 derivative	7
JD26186	*bioC::KanR*	*E. coli* KP7600 derivative with disrupted *bioC* gene	NBRP, Japan
JD26186 *birA::CAT*	*bioC::KanR birA::CAT*	JD28186 with CAT insertion in endogenous Ec*birA*	6
JD26186 *birA::CAT-SabioO-SaBirA*	*bioC::KanR birA::CAT (SabioO-lacZ)HK(placUV5-SaBirA)*	JD26186 birA::CAT with with *Sa*BioO-lacZ reporter integrated at HK022 att site and placUV5-*Sa*BirA integrated at lambda att site.	6
JD26186 *birA::CAT-SabioY-SaBirA*	*bioC::KanR birA::CAT (SabioY-lacZ)HK (placUV5-SaBirA)*	JD26186 birA::CAT with *Sa*BioY-lacZ reporter integrated at HK022 att site and placUV5-*Sa*BirA integrated at lambda att site.	6
JD26186 *birA::CAT-yhfST-SaBirA*	*bioC::KanR birA::CAT (yHFT-lacZ)HK (placUV5-SaBirA)*	JD26186 birA::CAT with yHFT-lacZ reporter integrated at HK022 att site and placUV5-*Sa*BirA integrated at lambda att site.	6
JD26186 *birA::CAT-SabioO-SaBirA-F123G*	*bioC::KanR birA::CAT (SabioO-lacZ)HK (placUV5-SaBirA-F123G)*	JD26186 birA::CAT with *Sa*BioO-lacZ reporter integrated at HK022 att site and plac-UV5-*Sa*BirA-F123G integrated at lambda att site.	this study
JD26186 *birA::CAT-SabioY-SaBirA-F123G*	*bioC::KanR birA::CAT (SabioY-lacZ)HK (placUV5-SaBirA-F123G)*	JD26186 birA::CAT with *Sa*BioY-lacZ reporter integrated at HK022 att site and plac-UV5-*Sa*BirA-F123G integrated at lambda att	this study
JD26186 *birA::CAT-yhfST-SaBirA-F123G*	*bioC::KanR birA::CAT (yHFT-lacZ)HK (placUV5-SaBirA-F123G)*	JD26186 birA::CAT with yHFT-lacZ reporter integrated at HK022 att site and placUV5-*Sa*BirA-F123G integrated at lambda att	this study

JD26186-*EcBioO-EcBirA*	*bioC::KanR (EcBioO-lacZ)HK*	JD26186 with *Ec*BioO-lacZ reporter chromosomally integrated at HK022 att site	[6]
JD26186 *birA::CAT-EcBioO-EcBirA-R119W*	*bioC::KanR birA::CAT (EcBioO-lacZ)HK (placUV5-EcBirA-R119W)*	JD26186 birA::CAT with *Ec*BioO-lacZ reporter and placUV5-*Ec*BirA-R119W integrated at lambda att	this study

Supporting Table S4: Oligomeric state validation by native nano-electrospray ionisation mass-spectroscopy (nESI-MS)

*Ec*BirA and *Sa*BirA were analyzed using nESI-MS under native conditions to determine the oligomeric state of the protein in the absence and presence of of biotin and MgATP. Holo-BirA was prepared by incubating the apo-purified proteins with 500 µM biotin and 1 mM MgATP, prior to buffer exchange for MS analysis. The measured molecular masses (Da) and the corresponding oligomeric states are outlined, along with the predicted masses (Da) for these states. All measured masses are within the acceptable error range of ~1% in 1 MDa [8].

BirA Sample	Measured MWs (Da)	Complex components	Calculated MWs (Da)
Apo-wild type *Ec*BirA	36203	Monomer	36192
Holo-wild type *Ec*BirA	36771<br><br>73559	Monomer, biotinyl-5′-AMP bound<br><br>Dimer, biotinyl-5′-AMP bound	36765<br><br>73530
Apo-R119W *Ec*BirA	36216	Monomer	36222
Holo-R119W *Ec*BirA	36783	Monomer, biotinyl-5′-AMP bound	36795
Apo-wild type *Sa*BirA	37892	Monomer	37892
Holo-wild type *Sa*BirA	38470<br><br>76925	Monomer, biotinyl-5′-AMP bound<br><br>Dimer, biotinyl-5′-AMP bound	38466<br><br>76931
Apo-F123G *Sa*BirA	37802	Monomer	37802
Holo-F123G *Sa*BirA	38381	Monomer, biotinyl-5′-AMP bound	38376

Supporting Table S5: Native nESI-MS measured masses for BirA-DNA complexes.

BirA and double-stranded oligonucleotide complexes were prepared prior to analysis by nESI-MS as described in materials and methods. The measured molecular weights (MW) for the species detected for each BirA-DNA complex were compared against the predicted sizes (calculated MW). Description of each species detected is outlined. The measured MWs were within the accepted mass differences of ~1% in 1 MDa [8].

BirA	Measured MW (Da)	Complex components	Calculated MW (Da)
Apo-*Ec*BirA (WT) + *Ec*bioO	30767	Free *Ec*bioO	30761
	36200	Apo-*Ec*BirA (WT) monomer	36192
	103138	2 Apo-*Ec*BirA (WT) subunits bound to *Ec*bioO	103145
Holo-*Ec*BirA (WT) + *Ec*bioO	30767	Free *Ec*bioO	30761
	72382	Apo-*Ec*BirA (WT) dimer	72384
	73522	Holo-*Ec*BirA (WT, biotinyl-5'-AMP bound) dimer	73530
	104272	2 Holo-*Ec*BirA (WT, biotinyl-5'-AMP bound) subunits bound to *Ec*bioO	104292
Apo-*Ec*BirA (R119W) + *Ec*bioO	30771	Free *Ec*bioO	30761
	36210	Apo-*Ec*BirA (R119W) monomer	36222
	103191	2 Apo-*Ec*BirA (R119W) subunits bound to *Ec*bioO	103205
Holo-*Ec*BirA (R119W) + *Ec*bioO	30768	Free *Ec*bioO	30761
	104348	2 Holo-*Ec*BirA (R119W, biotinyl-5'-AMP bound) subunits bound to *Ec*bioO	104351
Apo-*Sa*BirA (WT) + *Sa*bioY	27050	Free *Sa*bioY	27053
	102819	2 Apo-*Sa*BirA (WT) subunits bound to *Sa*bioY	102837
Holo-*Sa*BirA (WT) + *Sa*bioY	27050	Free *Sa*bioY	27053
	103989	2 Holo-*Sa*BirA (WT, biotinyl-5'-AMP bound) subunits bound to *Sa*bioY	103984
Apo-*Sa*BirA (F123G) + *Sa*bioY	27061	Free *Sa*bioY	27053
	102653	2 Apo-*Sa*BirA (F123G) subunits bound to *Sa*bioY	102657
Holo-*Sa*BirA (F123G) + *Sa*bioY	27063	Free *Sa*bioY	27053
	103806	2 Holo-*Sa*BirA (F123G, biotinyl-5'-AMP bound) subunits bound to *Sa*bioY	103804

Supporting Table S6: Native nESI-MS analysis for *Sa*BirA in complex with an oligonucleotide containing two mutated half sites.

*Sa*BirA was tested for binding specificity by incubating the repressor with a double-stranded oligonucleotide containing two mutated recognition sequences. The sequence of the oligonucleotide is shown in Supporting Figure S3. Measured molecular weights (MW) for the species detected were compared against the predicted sizes (calculated MW). Description of each species is outlined. The measured MWs were within the accepted mass differences of ~1% in 1 MDa [8].

BirA	Measured MW (Da)	Complex components	Calculated MW (Da)
Apo-*Sa*BirA (WT) + mutated oligonucleotide	23897	Free mutated oligonucleotide	23886
	37887	Apo-*Sa*BirA (WT) monomer	37892
Holo-*Sa*BirA (WT) + mutated oligonucleotide	23896	Free mutated oligonucleotide	23886
	37902	Apo-*Sa*BirA (WT) monomer	37892
	38474	Holo-*Sa*BirA (WT, biotinyl-5'-AMP bound) monomer	38466
Apo-*Sa*BirA (F123G) + mutated oligonucleotide	23897	Free mutated oligonucleotide	23886
	37817	Apo-*Sa*BirA (F123G) monomer	37802
Holo-*Sa*BirA (F123G) + mutated oligonucleotide	23889	Free mutated oligonucleotide	23886
	38370	Holo-*Sa*BirA (F123G, biotinyl-5'-AMP bound) monomer	38376

Supporting Table S7: Native nESI-MS measured masses for *Sa*BirA in complex with a *Sa*bioO oligonucleotide containing a single mutated half-site.

Complexes of *Sa*BirA and double-stranded *Sa*bioO oligonucleotides with the first half-site recognition sequence mutated were prepared as described in materials and methods. The sequence of the oligonucleotide is shown in Supporting Figure S4A. Measured molecular weights (MW) of the species detected were compared against the predicted sizes (calculated MW). Description of each species is outlined. The measured MWs were within the accepted mass differences of ~1% in 1 MDa [8].

<table>
<tr><th>BirA</th><th>Measured MW (Da)</th><th>Complex components</th><th>Calculated MW (Da)</th></tr>
<tr><td rowspan="3">Apo-SaBirA (WT) + 1 half-site mutated SabioO</td><td>23901</td><td>Free oligonucleotide</td><td>23884</td></tr>
<tr><td>37881</td><td>Apo-SaBirA (WT) monomer</td><td>37892</td></tr>
<tr><td>61773</td><td>1 Apo-SaBirA (WT) subunit bound to 1 half-site mutated SabioO</td><td>61776</td></tr>
<tr><td rowspan="5">Holo-SaBirA (WT) + 1 half-site mutated SabioO</td><td>23888</td><td>Free oligonucleotide</td><td>23884</td></tr>
<tr><td>37899</td><td>Apo-SaBirA (WT) monomer</td><td>37892</td></tr>
<tr><td>38475</td><td>Holo-SaBirA (WT, biotinyl-5'-AMP bound) monomer</td><td>38466</td></tr>
<tr><td>76942</td><td>Holo-SaBirA (WT, biotinyl-5'-AMP bound) dimer</td><td>76931</td></tr>
<tr><td>100811</td><td>2 Holo-SaBirA (WT, biotinyl-5'-AMP bound) bound to 1 half-site mutated SabioO</td><td>100815</td></tr>
<tr><td rowspan="3">Apo-SaBirA (F123G) + 1 half-site mutated SabioO</td><td>23891</td><td>Free oligonucleotide</td><td>23884</td></tr>
<tr><td>37812</td><td>Apo-SaBirA (F123G) monomer</td><td>37802</td></tr>
<tr><td>61687</td><td>1 Apo-SaBirA (F123G) subunit bound to 1 half-site mutated SabioO</td><td>61686</td></tr>
<tr><td rowspan="4">Holo-SaBirA (F123G) + 1 half-site mutated SabioO</td><td>23893</td><td>Free oligonucleotide</td><td>23884</td></tr>
<tr><td>38385</td><td>Holo-SaBirA (F123G, biotinyl-5'-AMP bound) monomer</td><td>38376</td></tr>
<tr><td>62268</td><td>1 Holo-SaBirA (F123G, biotinyl-5'-AMP bound) subunit bound to 1 half-site mutated SabioO</td><td>62260</td></tr>
<tr><td>100638</td><td>2 Holo-SaBirA (F123G, biotinyl-5'-AMP bound) subunits bound to 1 half-site mutated SabioO</td><td>100635</td></tr>
</table>

References:

1 Wood, Z. A., Weaver, L. H., Brown, P. H., Beckett, D. & Matthews, B. W. Co-repressor induced order and biotin repressor dimerization: a case for divergent followed by convergent evolution. *J Mol Biol* **357**, 509-523, doi:10.1016/j.jmb.2005.12.066 (2006).

2 Soares da Costa, T. P. *et al.* Selective inhibition of biotin protein ligase from Staphylococcus aureus. *J Biol Chem* **287**, 17823-17832, doi:10.1074/jbc.M112.356576 (2012).

3 Soares da Costa, T. P. *et al.* Dual roles of F123 in protein homodimerization and inhibitor binding to biotin protein ligase from Staphylococcus aureus. *Mol Microbiol* **91**, 110-120, doi:10.1111/mmi.12446 (2014).

4 St-Pierre, F. *et al.* One-step cloning and chromosomal integration of DNA. *ACS Synthet. Biol.* **2**, 537-541, doi:10.1021/s400021j (2013).

5 Cui, L., Murchland, I., Dodd, I. B. & Shearwin, K. E. Bacteriophage lambda repressor mediates the formation of a complex enhancer-like structure. *Transcription* **4**, 201-205 (2013).

6 Satiaputra, J. *et al.* Biotin-mediated growth and gene expression in *Staphylococcus aureus* is highly responsive to environmental biotin. *Appl Microbiol Biotechnol* **102**, 3793-3803, doi:10.1007/s00253-018-8866-z (2018).

7 Miki, T., Yamamoto, Y. & Matsuda, H. A novel, simple, high-throughput method for isolation of genome-wide transposon insertion mutants of Escherichia coli K-12. *Methods Mol Biol* **416**, 195-204, doi:10.1007/978-1-59745-321-9_13 (2008).

8 Benesch, J. L. P. & Ruotolo, B. T. Mass Spectrometry: an Approach Come-of-Age for Structural and Dynamic Biology. *Curr Opin Struct Biol* **21**, 641-649 (2011).
